# Porphyrin-Loaded Lignin Nanoparticles Against Bacteria: A Photodynamic Antimicrobial Chemotherapy Application

**DOI:** 10.3389/fmicb.2020.606185

**Published:** 2020-11-17

**Authors:** Nidia Maldonado-Carmona, Guillaume Marchand, Nicolas Villandier, Tan-Sothea Ouk, Mariette M. Pereira, Mário J. F. Calvete, Claude Alain Calliste, Andrzej Żak, Marta Piksa, Krzysztof J. Pawlik, Katarzyna Matczyszyn, Stéphanie Leroy-Lhez

**Affiliations:** ^1^PEIRENE Laboratory, Faculty of Sciences and Techniques, University of Limoges, Limoges, France; ^2^Laboratory of Catalysis and Fine Chemistry, Department of Chemistry, University of Coimbra, Coimbra, Portugal; ^3^PEIRENE Laboratory, Faculty of Pharmacy, University of Limoges, Limoges, France; ^4^Electron Microscopy Laboratory, Wrocław University of Science and Technology, Wrocław, Poland; ^5^Institute of Immunology and Experimental Therapy, Polish Academy of Sciences, Wrocław, Poland; ^6^Advanced Materials Engineering and Modelling Group, Faculty of Chemistry, Wrocław University of Science and Technology, Wrocław, Poland

**Keywords:** tetrapyrrolic compounds, valorized lignin, nanoparticles, photodynamic antimicrobial therapy, antimicrobial alternatives

## Abstract

The need for alternative strategies to fight bacteria is evident from the emergence of antimicrobial resistance. To that respect, photodynamic antimicrobial chemotherapy steadily rises in bacterial eradication by using light, a photosensitizer and oxygen, which generates reactive oxygen species that may kill bacteria. Herein, we report the encapsulation of 5,10,15,20-tetrakis(4-hydroxyphenyl)-21H,23H-porphyrin into acetylated lignin water-dispersible nanoparticles (**THPP@AcLi**), with characterization of those systems by standard spectroscopic and microscopic techniques. We observed that **THPP@AcLi** retained porphyrin’s photophysical/photochemical properties, including singlet oxygen generation and fluorescence. Besides, the nanoparticles demonstrated enhanced stability on storage and light bleaching. **THPP@AcLi** were evaluated as photosensitizers against two Gram-negative bacteria, *Escherichia coli* and *Pseudomonas aeruginosa*, and against three Gram-positive bacteria, *Staphylococcus aureus*, *Staphylococcus epidermidis*, and *Enterococcus faecalis*. **THPP@AcLi** were able to diminish Gram-positive bacterial survival to 0.1% when exposed to low white LED light doses (4.16 J/cm^2^), requiring concentrations below 5 μM. Nevertheless, the obtained nanoparticles were unable to diminish the survival of Gram-negative bacteria. Through transmission electron microscopy observations, we could demonstrate that nanoparticles did not penetrate inside the bacterial cell, exerting their destructive effect on the bacterial wall; also, a high affinity between acetylated lignin nanoparticles and bacteria was observed, leading to bacterial flocculation. Altogether, these findings allow to establish a photodynamic antimicrobial chemotherapy alternative that can be used effectively against Gram-positive topic infections using the widely available natural polymeric lignin as a drug carrier. Further research, aimed to inhibit the growth and survival of Gram-negative bacteria, is likely to enhance the wideness of acetylated lignin nanoparticle applications.

## Introduction

Antimicrobial resistance (AMR) upraise is one of the greatest challenges that modern medicine and chemistry are facing. After the “golden age of antibiotics,” the decay on the discovery rate of new and more efficient molecules was conjugated with the appearance of antimicrobial-resistant strains. AMR alone is expected to cause 10 million deaths by 2050, with an accumulative cost of 100 trillion USD (O’Neill, 2016). However, this prediction only accounts for developed countries, and this number is expected to be higher and still to be determined, with the greatest impact on developing countries. The World Health Organization has devised a global action plan (WHO, 2015) emphasizing the necessity to find new antimicrobial alternatives and to improve disinfection processes, while exploring therapeutic approaches that are less prone to generate resistance (Lewis, 2013; Regiel-Futyra et al., 2017).

In that respect, photodynamic therapy (PDT) has revealed to be a suitable alternative. PDT is the conjugation of light and a photosensitizer molecule, generating reactive oxygen species (ROS) from either molecular oxygen in the media (Type II mechanism) or a substrate (Type I mechanism) (Josefsen and Boyle, 2012). When these ROS are directed against microorganisms, the process is addressed as photodynamic antimicrobial chemotherapy (PACT) (Wainwright, 2019). These *in situ*-generated ROS are able to destroy biomacromolecules, including proteins, membrane lipids, and nucleic acids, through a non-specific target mechanism (Wainwright et al., 2017). Commonly in PDT addressed against cancer, desired photosensitizers are molecules with strong absorption bands near the infrared range (700–900 nm), which coincides with the skin transparent wavelengths, and permit light to reach deeper through the skin (Josefsen and Boyle, 2012). In contrast, most of the applications of PACT are at surfaces or topic applications, thus photosensitizing molecules are not limited to absorption in the infrared range. Recent PACT applications have been developed for usage under white light (Nzambe Ta keki et al., 2016; Ringot et al., 2018; Aroso et al., 2019; Khaldi et al., 2019), blue light (Buchovec et al., 2016), and even solar light (Jia et al., 2019). Currently, PACT applications are actively pursued by several research groups, with applications in dentistry as a complement of systemic antibiotic treatments (de Freitas et al., 2016; Bechara Andere et al., 2018), as a non-invasive treatment against *Helicobacter pylori* (Baccani et al., 2019), and even as an environment-friendly alternative for active food packaging (i.e., biodegradable coatings for strawberries disinfection), food disinfection (i.e., curcumin derivatives for lettuce and mung beans disinfection), and other agronomical applications (i.e., porphyrinic insecticides and pesticides) (Riou et al., 2014; Buchovec et al., 2016; Glueck et al., 2017; Martinez et al., 2017).

In parallel, organic matrices (e.g., cellulose, chitosan, cyclodextrin) have been used for the transport and encapsulation of small molecules, with some examples of conjugating photosensitizers, enabling bacterial eradication and, in some cases, demonstrating a synergistic effect with the organic matrix (Hsieh et al., 2019; Maldonado-Carmona et al., 2020). In this regard, an organic matrix that has been neglected is lignin. Lignin is a natural aromatic polymer, representing up to 20–35% of the total lignocellulosic biomass, and it is usually a by-product of the paper industry. It is a polymer of p-coumaryl alcohol, coniferyl alcohol, and sinapyl alcohol units, whose proportions vary according to its botanic origin (Faix, 1991). Due to its chemical nature, it can withstand several chemical modifications, either through the creation of new chemically active sites or through the substitution of the already available ones (Calvo-Flores and Dobado, 2010; Duval and Lawoko, 2014; Wang et al., 2016; Figueiredo et al., 2018; Marchand et al., 2018). In addition to chemical modifications, different methods for the preparation of lignin-based nanomaterials had been developed. One of the main applications given to these nano-objects is the loading and release of active substances.

Understandably, lignin has not been widely used as a photosensitizing molecule’s vehicle mainly due to the widely known antioxidant activity of lignin (Ponomarenko et al., 2015; Yang et al., 2016). To the best of our knowledge, only one approach is reported in literature using lignin-coated noble metal nanoparticles for *Staphylococcus aureus* and *Escherichia coli* photo-induced disinfection (Rocca et al., 2018). Another report has been found where lignin was linked to a phenyl porphyrin, resulting in a biopolymer with increased fluorescence, but no photodynamic approach was implied (Tse et al., 2019). Besides, lignin nanoparticles are demonstrated to be innocuous to *Chlamydomonas reinhardtii*, an aquatic microorganism, and to *Saccharomyces cerevisiae*, a eukaryotic cell model (Frangville et al., 2012). Additionally, they are demonstrated to be innocuous against Caco-2 cells (Alqahtani et al., 2019), a human colon carcinoma cell line. Among all the possible lignin modifications, lignin acetylation is widely described in the literature (Qian et al., 2014b). For instance, recent reports have demonstrated that acetylated Kraft and Organosolv lignins (**AcLi**) work as weak photosensitizers (Marchand et al., 2018) and that **AcLi** is also able to form spherical nanoparticles (Qian et al., 2014a, b; Marchand et al., 2020), further demonstrating the capability to transport active molecules (Zhou et al., 2019; Marchand et al., 2020).

Considering all the above, here we report the encapsulation of commercial 5,10,15,20-tetrakis(4-hydroxyphenyl)-21*H*,23*H-*porphyrin (**THPP**) inside acetylated lignin nanoparticles **(@AcLi**). The nanoparticles were characterized through transmission electron microscopy (TEM), dynamic light scattering (DLS), zeta potential, UV-vis absorption and fluorescence, and electron paramagnetic resonance (EPR) in order to evaluate their capacity to generate singlet oxygen. The nanoparticles were tested against three Gram-positive bacteria, *S. aureus* CIP 76.25, *Staphylococcus epidermidis* CIP 109562, and *Enterococcus faecalis* CIP 76.1170, and against two Gram-negative bacteria, *E. coli* CIP 53.126 and *Pseudomonas aeruginosa* CIP 76.110, under white LED light irradiation. Additionally, **THPP**-loaded **@AcLi** (**THPP@AcLi**) were evaluated for their stability over long storage periods, and their properties were assessed at different pH ranges.

## Materials and Methods

### Materials and Microbiological Strains

Kraft lignin was kindly donated by the Université du Québec à Trois-Rivières, Canada. **THPP**, acetic anhydride, dry pyridine, and other reagents were purchased at Sigma-Aldrich (Lyon, France) and used as received, without further purification. *E. coli* CIP 53.126, *E. faecalis* CIP 76.1170, *P. aeruginosa* CIP 76.110, *S. aureus* CIP 76.25, and *S. epidermidis* CIP 109562 were obtained from the Institute Pasteur Collection (Institute Pasteur, Paris, France). All bacterial strains were kept frozen as small aliquots (100 μl), at −78°C, with glycerol 50% as cryopreservant. A whole aliquot was used for each culture, avoiding defrosting of the other samples. *P. aeruginosa* was grown in Luria–Bertani (LB) broth (tryptone 10 g/L, sodium chloride 10 g/L, yeast extract 5 g/L), while all the other bacterial strains were routinely growth at trypto-casein soy medium (TS, Biokar; tryptone 17 g/L, papaic digest of soybean meal 3 g/L, glucose 2.5 g/L, dipotassium phosphate 2.5 g/L, sodium chloride 2 g/L), prepared as a broth (LBB and TSB) or as a solid media (LBA and TSA; 1.7% agar) according to standard procedures. Saline solution (0.9% NaCl) and phosphate buffer pH 7 (PB pH 7, NaH_2_PO_4_ 6.045 g/L, Na_2_HPO_4_ 10.5 g/L) were routinely prepared and sterilized.

### Preparation of Acetylated Lignin

AcLi was prepared according to previous publications (Marchand et al., 2018). A kraft lignin solution (50 mg/ml) was prepared in an acetic anhydride/dry pyridine (1:1) mixture and stirred at 25°C, under a calcium chloride (CaCl_2_) trap, for 48 h. Then, the reaction mixture was poured onto 500 ml distilled water, and the precipitate was filtrated, dissolved on chloroform, and washed three times with distilled water. The organic phase was dried with MgSO_4_ and evaporated to dryness.

### Acetylated Lignin Characterization

Acetylated lignin was dissolved in acetonitrile, and its UV-vis absorption spectrum was recorded on a spectrophotometer Specord 210 Lambda (Analytik Jena) on quartz cells. FT-IR spectrum of materials was obtained using a Frontier PerkinElmer spectrometer in the attenuation total reactance analysis mode. Spectra were collected between 600 and 4,000 cm^–1^ after placing the pure product on a diamond crystal plate.

### Preparation and Quantification of Acetylated Lignin Nanoparticles

Nanoparticles were prepared as previously described (Figueiredo et al., 2017). Acetylated lignin nanoparticles were prepared starting from an acetylated lignin solution (2 mg/ml) in acetone. For **THPP** encapsulation, **THPP** (0.2 mg/ml) was added in the acetonic solution. The AcLi solution was dialyzed on a regenerated cellulose membrane rod (Fisherbrand, 12–14 kDa) against distilled water for 24 h. After dialysis, nanoparticles were centrifuged at 10,000 × *g* for 1 h. Then, nanoparticles were washed with distilled water and centrifuged again. Finally, nanoparticles were suspended in distilled water and stored for further use. Routinely, after the harvest of **THPP@AcLi**, a small amount of nanoparticles was dissolved in acetone, and **THPP** quantification was done using the Soret band absorption (λ_*max*_ 419 nm, ε = 388,500 L/mol cm). Similarly, nanoparticles were dissolved in acetonitrile for AcLi quantification. The volume of dissolved nanoparticles was always below 3%, regarding the final volume on organic solvent. For **THPP@AcLi** analysis in aqueous media, nanoparticles were diluted on an appropriated buffer and their spectra were recorded. Spectra were collected between 200 and 800 nm. The encapsulation rate was calculated, as the ratio of the amount of **THPP** inside the nanoparticles, to the initial amount (Eq. 1):

(1) $$Encapsulationrate\left(\%\right)=\frac{C_{THPP}V_{Np}MW_{THPP}}{THPP_{i}}\times 100$$

where C**_*THPP*_** was the observed concentration of **THPP** in the final volume of nanoparticles (V_*Np*_), considering the molecular weight of **THPP** (MW**_*THPP*_**) and the initial mole number of **THPP** (**THPP**_*i*_).

### Apparent Size and Zeta Potential Analysis

Nanoparticle size was analyzed through DLS on a Zetasizer Nano-ZS (Malvern Instrument). Three measurements were performed on each sample at 20°C using a light scattering angle of 173° and a refractive index of 1.59 for lignins. Nanoparticles were diluted on distilled water for each DLS determination. The obtained DLS raw data were fit to a Gaussian model, excluding the values with less than 1% of presence. The obtained data were validated through the analysis of their R square coefficient and through the analysis of the residuals with a D’Agostino–Pearson Omnibus K2 test. With this statistical approach, we obtained the mean size (geometrical mean) and the standard deviation (σ), which allowed us to approximate the range where 95% of the nanoparticles were found (2σ). Zeta potential was obtained with the same equipment, and nanoparticles were diluted on an appropriated aqueous solution for each determination.

### Transmission Electron Microscopy Observations

The samples were observed using the TEM, model H-800 (Hitachi), using an accelerating voltage of 150 kV. For nanoparticle imaging, a dense suspension of nanoparticles in water was used. Two microliters of each sample were deposited on carbon on the copper grid. The excess of liquid was carefully blotted with a filter paper and air-dried for 1 h. For bacterial and interaction observations, an overnight culture of *S. aureus* in TSB was washed with PB pH 7.4 (5,000 × *g*, 5 min) three times. Then, bacteria were carefully suspended on a minimal volume of buffer. After deposition of the 2 μl of the sample on grid and blotting, the samples were fixed and negative stained with 2 μl drop of 2% uranyl acetate deposited on the grid. The stain was blotted after 60 s, and the samples were air-dried for 1 h. For the interaction observations, 150 μl from the bacterial suspension was mixed with 150 μl of the nanoparticle suspension. Light irradiation was done with the 2 μl mixed sample on the TEM grid, under an incandescent lamp, with light irradiation of around 2,500 lux for 5 min. After that, the samples were blotted and fixed as described above. The scheme of the preparation process is shown in Supplementary Material 1. For nanoparticle size determination, ImageJ Fiji (Schindelin et al., 2012; Schneider et al., 2012) software was used (Thresholding default, size 0.01–1.00 μm^2^, circularity 0.06–1.00).

### Stability of 5,10,15,20-Tetrakis(4-Hydroxyphenyl)-21H,23H-Porphyrin Inside Acetylated Lignin Nanoparticles

The stability of the encapsulation was tested over time. For this, a suspension of **THPP@AcLi** at 100 μM was prepared and divided in small 500-μl fractions, which were stored at 25°C in the dark. When analyzed, samples were centrifuged (10,000 × *g*, 30 min) and the supernatant was retired. The pelleted nanoparticles were resuspended in distilled water, and both nanoparticles and supernatants were analyzed by UV-vis absorption spectroscopy. The stability of the nanoparticles was followed through the changes of the Soret band absorption (λ_*max*_ 430 nm) over time in both nanoparticles and supernatants.

### Singlet Oxygen Detection by Electron Paramagnetic Resonance

Measurements were recorded as described elsewhere (Riou et al., 2014). The samples were exposed to a 20 W halogen lamp, with a light irradiation of 20,000 lux. The intensity of illumination was measured by a lux meter (Digital Lux Tester YF-1065). EPR spectra were recorded with a Bruker Model ESP300E spectrometer operating at room temperature. Routinely, a fresh solution of 25 mM 2,2,6,6-tetramethylpiperidine (**TEMP**) was prepared in phosphate buffer pH 7.4. Acetylated lignin nanoparticle suspension was prepared at a concentration of 4 mg/ml of AcLi, while **THPP@AcLi** suspension was diluted at 120 μM of **THPP** or 0.2 mg/ml of AcLi. For singlet oxygen detection, 50 μl of the fresh **TEMP** solution were mixed with 50 μl of the nanoparticle suspension. The solution obtained was immediately transferred into quartz capillaries (100 μl) and placed at 20 cm from the source of illumination with a light intensity of 270 μE/(s m^2^) during periods of 5 min. A dark control was prepared, and rose Bengal in dimethylformamide (DMF) was used as a standard. EPR spectra were performed under the following conditions: modulation frequency, 100 kHz; microwave frequency, 9.78 GHz; microwave power, 4 mW; modulation amplitude, 0.987 G; time constant, 10.24 ms; scans number, 2.

### Fluorescence Quantum Yield

Fluorescence quantum yield was calculated as described elsewhere (Vinagreiro et al., 2020). The fluorescence emission spectra were recorded in a Horiba Scientific Spectrofluorometer Fluoromax-4. The spectra were collected from 550 up to 800 nm using standard quartz cuvettes of 1 cm of optical path. Fluorescence quantum yields (Φ_*F*_) were obtained by comparing the area of integrated fluorescence of the samples (F_*s*_) with that of the reference (F_*ref*_) compound, with known Φ_*F*_, corrected by the absorption of sample (A_*s*_) and reference (A_*ref*_) at the excitation wavelength and by the refractive index of the solvents used for the sample (η_*s*_) and reference (η_*ref*_) solutions (Eq. 2).

(2) $$\Phi_{F}=\Phi_{F}^{ref}\frac{F_{s}A_{ref}\eta_{s}^{2}}{F_{ref}A_{s}\eta_{ref}^{2}}$$

Tetraphenylporphyrin (**TPP**) in toluene (Φ*_*F*_* ref = 0.11) was used as standard (Pineiro et al., 1998). The absorbance of the sample at the excitation wavelength was around 0.01.

### Photobleaching Quantum Yield

Photobleaching experiments were done at similar conditions as those carried away for the microbiological experiments and following the procedure stated elsewhere (Vinagreiro et al., 2020). **THPP@AcLi** were diluted to a final concentration of 10 μM in PB pH 7. A volume of 200 μl (V_*irr*_) was deposited on a flat-bottom 96-well plate (l = 1 cm) and irradiated, ensuring that all the light went through the solution. The samples were irradiated for a time Δ_*t*_ using a white LED light with emission (λ_*Em*_) at 447 nm and output power P_0_ of 1 mW. The actual light power absorbed was determined for each compound and properly taken into account in the calculation of the photobleaching quantum yield, as described in the Supplementary Material 2 (Schaberle, 2018). Photobleaching quantum yield (Φ_*pb*_) is defined as the ratio between the rate of disappearance of photosensitizer molecules (*v*_*d*_) and the rate of absorption of photons (*v*_*P*_) (Eq. 3).

(3) $$\Phi_{Pb}=\frac{v_{d}}{v_{p}}=\frac{V_{irr}N_{A}hc\Delta A_{Soret}}{\varepsilon_{Soret}l\lambda_{Em}P\left(1-10^{-A_{0}}\right)\Delta_{t}}$$

where A_0_ is the initial absorption at the Soret band. The Soret band absorbance was found to decrease, and its decay was followed during the light exposure.

### Photodynamic Antimicrobial Chemotherapy Bacteriostatic Effect

The bacteriostatic effect was evaluated against planktonic bacteria in the middle of the exponential phase of growth. An aliquot of bacteria was inoculated in 5 ml of TSB or LB and incubated for 16 h, 37°C, 100 rpm. The OD_600_ was measured for the resulting culture, and it was diluted at an OD_600_ = 0.05 in 5 ml of fresh TSB or LB. Bacteria subcultures were incubated under the same previous conditions, during 2 h for *E. coli*, and 3 h for the other bacteria. Bacteria were washed with sterile PB pH 7 (5,000 × *g*, 5 min), and 100 μl were diluted in 10 ml of PB pH 7 for a final concentration of ∼10^6^ CFU/ml. Onto a 96-well plate, a volume of 50 μl of bacterial suspension was mixed with 50 μl of a solution with **THPP@AcLi** or **@AcLi**, at geometrically decreasing concentrations, ranging from 50 to 0.010 μM and 1.6 mg/ml to 6.25 μg/ml, respectively. The plate was irradiated under white LED light (1.2 mW/cm^2^) for 1 h. A volume of 100 μl of TSB media was added to each well, and the initial OD_595_ (S_*i*_) was acquired using an iMark multiplate reader (Bio-Rad). The plate was incubated at 37°C in the dark for 6 h, and then its OD_595_ was measured again (S_*t*_). Appropriated controls were prepared, a sample without bacteria and treatment (B_0_) was used as a blank, while a sample without treatment was used as growth control and addressed as concentration zero (G_*i*_) and followed over time (G_*t*_). Normalized bacterial growth (G_*B*_) was obtained according to Eq. 4.

(4) $$G_{B}=\frac{S_{t}-S_{i}}{G_{t}-G_{i}}\;$$

A second 96-well plate was prepared, with bacteria and nanoparticles at the same concentrations and conditions and kept away from the light. Bacterial growth was allowed and monitored as the light-irradiated plate, becoming the dark control.

### Photodynamic Antimicrobial Chemotherapy Bactericidal Effect

The bactericidal effect was evaluated against planktonic bacteria in the middle of the exponential phase of growth. An aliquot of bacteria was inoculated in 5 ml of TSB or LB and incubated for 16 h, 37°C, 100 rpm. The OD_600_ was measured and diluted at an OD_600_ = 0.05 in 5 ml of fresh TSB or LB. Bacteria subcultures were incubated under the same previous conditions for 2 h for *E. coli* and 3 h for the other bacteria. Bacteria were washed with sterile PB pH 7 (5,000 × *g*, 5 min) and suspended in 10 ml of PB pH 7 for a final concentration of ∼10^8^ CFU/ml. Onto a 96-well plate, a volume of 50 μl of bacterial suspension was mixed with 50 μl of a solution with **THPP@AcLi**, at geometrically decreasing concentrations, ranging from 2.5 to 0.010 μM, while **@AcLi** was only tested at 1.6 mg/ml, the highest concentration tested at the PACT bacteriostatic effect. Appropriated controls were prepared; a sample without nanoparticle treatment was used as a survival control. The plate was irradiated under white LED light (1.2 mW/cm^2^) for 1 h. Besides, a second identical plate was prepared and kept away from light. Then, the solution on the wells was serially diluted on 900 μl of saline solution, and 50 μl were spread on TSA or LBA plates using an automatic plater EasySpiral (Interscience). Petri dishes were incubated at 37°C, in the dark, for 16 h. Colony-forming units (CFUs) were counted using a colony counter Scan 100 (Interscience). Bacterial survival was calculated, comparing the number of viable bacteria after the treatment (CFU/ml_*Treatment*_) with the number of viable bacteria without treatment (CFU/ml_*Control*_) (Eq. 5).

(5) $$Bacterialsurvival\left(\%\right)=\frac{CFU/mL_{Treatment}}{CFU/mL_{Control}}\times 100$$

### Statistical Analysis

Experiments were performed at least in triplicate. The results were analyzed with GraphPad Prism 6.01. Biological data were analyzed with a two-way ANOVA using a Sidak’s test for multiple comparisons with 95% of the cohort. The obtained DLS raw data were fit to a Gaussian model, excluding the values with less than 1% of presence. The obtained data were validated through the analysis of their R square coefficient and through the analysis of the residuals with a D’Agostino–Pearson Omnibus K2 test. With this statistical approach, we obtained the mean size (geometrical mean) and the standard deviation (σ), which allowed us to approximate the range of size that have 95% of the nanoparticles (D_95_).

## Results

### Preparation of Acetylated Lignin Nanoparticles and Their Physicochemical Characterization

**FIGURE 1:**
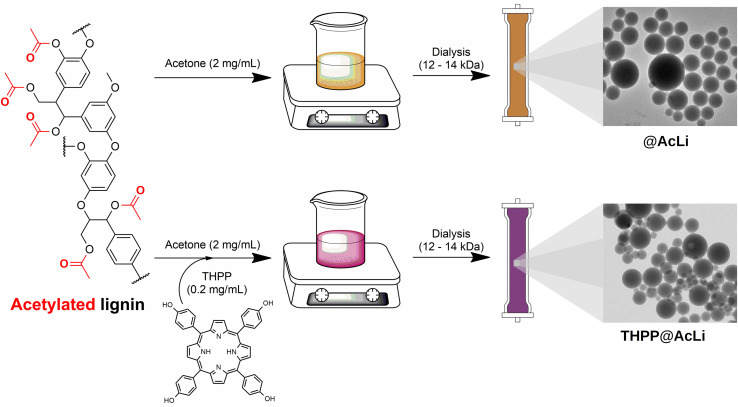
Preparation of acetylated lignin nanoparticles **(@AcLi**) and 5,10,15,20-tetrakis(4-hydroxyphenyl)-21*H*,23*H-*porphyrin (**THPP**)-loaded **@AcLi** (**THPP@AcLi**).

AcLi was prepared as previously described (Marchand et al., 2018), and their chemical properties are discussed in Supplementary Material 3. Acetylated lignin nanoparticles were prepared as previously described (Figueiredo et al., 2017; Marchand et al., 2020) from an acetone solution (Figure 1). **THPP** encapsulation was done at similar conditions, through the addition of **THPP** into the acetone solution. In both cases, the acetone solution was dialyzed against water. The thereof obtained nanoparticles were centrifuged and suspended in distilled water.

**FIGURE 2:**
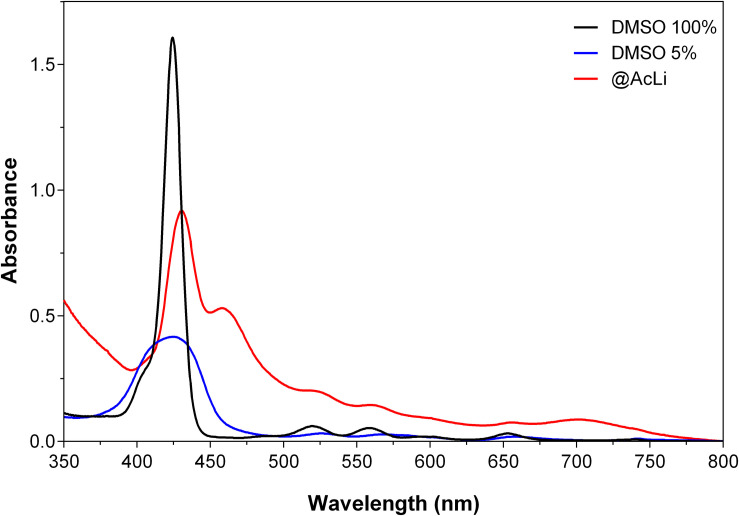
Comparison of the UV-vis spectra of 5,10,15,20-tetrakis (4-hydroxyphenyl)-21*H*,23*H-*porphyrin **(THPP)** 5 μM in DMSO (DMSO 100%) in a mixture of PB pH 7 and DMSO 5% (DMSO 5%) and as **THPP**-loaded acetylated lignin nanoparticles **(THPP@AcLi)**, suspended in PB pH 7.

**THPP@AcLi** suspension was analyzed with UV-vis absorption spectroscopy (Figure 2), and the spectroscopic data were summarized in Table 1. Due to the low aqueous solubility of **THPP**, the UV-vis spectrum of **THPP@AcLi** was compared with the spectra of **THPP** in dimethylsulfoxide (DMSO) and in a mixture of PB and DMSO (95/5 v/v), with the last simulating biological aqueous conditions. **THPP@AcLi** kept the typical porphyrin UV-vis absorption profile: one intense Soret band and four Q-bands at higher wavelengths. When compared with **THPP**, a red-shift of the Soret band in **THPP@AcLi** (λ = 430.5 nm) was observed compared with the observed Soret band in pure DMSO (λ = 424.5 nm). The observed red-shift and diminished absorbance that occurred were due to a solvatochromic effect and/or π-π interactions with the lignin aromatic core. Additionally, these features could also be due to the formation of **THPP** J-aggregates, as it has been previously described that, in the presence of water, **THPP** aggregates show a red-shifted Soret band with a diminished absorbance (Zannotti et al., 2018). **THPP** proneness to aggregate in aqueous medium was further corroborated with the observed wide and diminished Soret band for **THPP** in 5% DMSO. In addition, the appearance of extra bands at 457 nm (B-band) and at 701.5 nm (Q_*c*_) indicates the presence of protonated **THPP** (**THPPH_2_^2+^**) species inside the nanoparticles (Zannotti et al., 2018; Leroy-Lhez et al., 2019). Moreover, it seems that encapsulation of **THPP** inside **AcLi** nanoparticles reduced the aggregated state when compared to **THPP** dissolved in a mixture of aqueous media/organic solvent, where we observed a broad Soret band with diminished absorption.

**TABLE 1:** Absorption bands of **THPP** 5 μM in DMSO 100%, PB and DMSO 5%, and as **THPP@AcLi** suspended in PB pH 7.

	Soret band		λ_max_ B	λ_max_ Q_1_	λ_max_ Q_2_	λ_max_ Q_3_	λ_max_ Q_4_	λ_max_ Q_*C*_
	λ_max_	ε_max_ (L/mol cm)						
DMSO	424.5	3.214 × 10^5^		519.5	558.5	594.5	653	
PB pH 7 DMSO 5%	424.5	8.320 × 10^4^		526	568.5	599.5	657	
PB pH 7 **@AcLi**	430.5	1.836 × 10^5^	457	516.5	559.5	596.5	656	701.5

***THPP,** 5,10,15,20-tetrakis(4-hydroxyphenyl)-21H,23H-porphyrin; **THPP@AcLi, THPP**-loaded acetylated lignin nanoparticles.*

**FIGURE 3:**
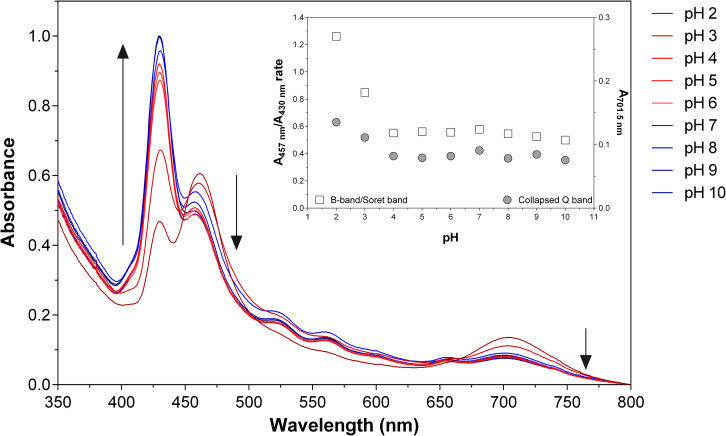
Absorption spectra of 5.87 μM 5,10,15,20-tetrakis(4-hydroxyphenyl)-21*H*,23*H-*porphyrin-loaded acetylated lignin nanoparticles **(THPP@AcLi)** suspensions at different pHs [0.1 M glycine-HCl buffer (pH 2 and pH 3), 0.1 M acetate buffer (pH 4 and pH 5), 0.1 M phosphate buffer (0.1 M pH 6, pH 7, and pH 8), 0.1 M glycine-NaOH buffer (pH 9 and pH 10)]. The black arrows depict the changes found through increasing the pH of the media. Inset, the ratio of absorbance values at 457 and 430.5 nm (squares, left axis) and the absorbance at 701.5 nm (circles, right axis) are depicted as a function of pH.

Given the presence of **THPPH_2_^2+^** species, further investigations on the effect of the pH on the loaded nanoparticles were deemed necessary. Thus, UV-vis absorption spectra of **THPP@AcLi** nanoparticles were recorded at several pH values using different buffers (0.1 M), as displayed in Figure 3. The **THPP@AcLi** UV-vis absorption profile remained stable at pH values above 4, as the proportion between the **THPP** and its protonated species **THPPH_2_^2+^** (expressed as ratio A_457_/A_430_) remained constant. Upon pH acidification, a change in the UV-vis absorption profile can be observed by a decrease of the absorbance at 430 nm with concomitant increase of the absorbance at 457 nm, reaching its maximum at pH 2, as depicted by the evolution of the ratio A_457_/A_430_ (Figure 3 inset, left axis). This, along with an increase of the absorbance at 701.5 nm, suggested an increased presence of protonated porphyrin **THPPH_2_^2+^** at values below pH 4. This experiment seems to corroborate the presence of both **THPP** and **THPPH_2_^2+^** species inside nanoparticles. Interestingly, neither at basic pH do the **THPPH_2_^2+^** related bands disappear, which either suggests the stability of the initial mixture or the solvent inaccessibility inside the nanoparticles. The last one has been previously analyzed through computational analysis on **@AcLi** formation, where nanoparticles have a lower solvent accessible surface area than lignin dissolved in organic solvent (Marchand et al., 2020). Nevertheless, **THPP** encapsulated inside **@AcLi** appears to be stable on a wide pH range (ca. 4–10), with its absorbance remaining unaffected by changes in the surrounding media.

Additionally, we could determine the amount of **THPP** encapsulated inside the nanoparticles through UV-vis quantification in acetone, as previously reported (Marchand et al., 2020). Our results demonstrated that up to 87.6% of the initial amount of **THPP** was encapsulated inside **@AcLi**; thus, the encapsulation process is not only sustainable but highly effective, allowing a good recovery of our photosensitizing molecule.

**FIGURE 4:**
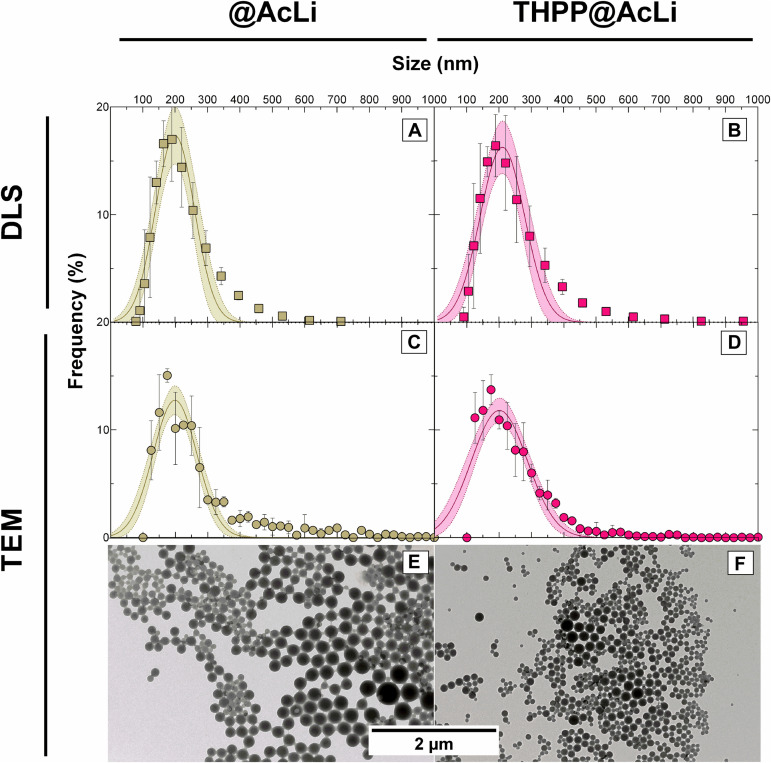
Size distribution of acetylated lignin nanoparticles **(@AcLi)** and 5,10,15,20-tetrakis(4-hydroxyphenyl)-21*H*,23*H-*porphyrin-loaded @AcLi (**THPP@AcLi)** measured through dynamic light scattering (DLS) **(A,B)** and through transmission electron microscopy (TEM) **(C,D)**. Also, the spherical shape of nanoparticles is observed for both **(E,F)**.

The size and shape of nanoparticles were also analyzed through two different methods, DLS and TEM. DLS indirectly permits to know the hydrodynamic size and the polydispersity index (PDI). The hydrodynamic size takes into account the presence of salts and water molecules surrounding the nanoparticles; thus, the size obtained is apparent and depends on the interaction of nanoparticles with the surrounding media. On the other hand, TEM observations directly analyze the size of opaque nanoparticles without taking into account the influence of the media; however, TEM observations need specialized software for image processing. In order to fully characterize the nanoparticles, both methods were compared for **THPP@AcLi** and **@AcLi** (Figure 4), with the results being summarized in Table 2. The results obtained were compared under the assumption that the nanoparticle populations follow a Gaussian distribution. DLS and TEM analysis obtained similar size values for both nanoparticles (approximately 200 nm). In both cases, **@AcLi** were slightly smaller than **THPP@AcLi**, by less than 10 nm. When analyzed, the PDI values obtained were below 0.2, which indicates that the degree of size dispersion was in the desired range of reported nanoparticles (Danaei et al., 2018), as high PDI values describe a wide distribution in the size of the population and is associated to flocculation of the samples. The wideness of the size of the nanoparticles was analyzed through the comparison of the range where 95% of the nanoparticles were found (D_95_). Both TEM and DLS analysis provided evidence that both populations had similar distributions, between 30 and 380 nm. These results were slightly different from those obtained by previous experiences (Marchand et al., 2020), where loaded nanoparticles have a smaller size than non-loaded nanoparticles. However, these differences could be due to differences in the workup of nanoparticles, as in the present work, nanoparticles are centrifuged at 10,000 × *g*.

**TABLE 2:** Size of nanoparticles determined by DLS and TEM, expressed as the Gaussian mean and the distribution of 95% of the nanoparticles (D_95_).

	DLS				TEM		
	Mean size (nm)	Range (D_95_)	R^2^ Gaussian model fitting	PDI	Mean size (nm)	Range (D_95_)	R^2^ Gaussian model fitting
**@AcLi**	199.6	79.02–320.18	0.9160	0.189	198.7	130.7–266.7	0.8410
**THPP@AcLi**	210.8	73.4–348.2	0.9154	0.176	200.6	114.16–287.4	0.8637

***@AcLi**, acetylated lignin nanoparticles; DLS, dynamic light scattering; PDI, polydispersity index; TEM, transmission electron microscopy;** THPP,** 5,10,15,20-tetrakis(4-hydroxyphenyl)-21H,23H-porphyrin; **THPP@AcLi, THPP**-loaded @AcLi.*

**FIGURE 5:**
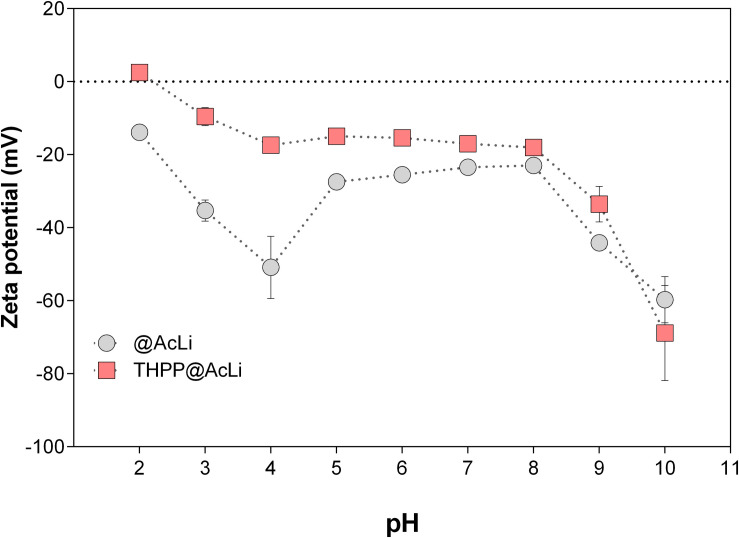
The pH effect on the zeta potential of acetylated lignin nanoparticles **(@AcLi)** and 5,10,15,20-tetrakis(4-hydroxyphenyl)-21 *H*,23*H-*porphyrin-loaded @AcLi **(THPP@AcLi)** [0.1 M glycine-HCl buffer (pH 2 and pH 3), 0.1 M acetate buffer (pH 4 and pH 5), 0.1 M phosphate buffer (0.1 M pH 6, pH 7, and pH 8), 0.1 M glycine-NaOH buffer (pH 9 and pH 10)].

In addition to size, another important parameter to characterize is the zeta potential. The zeta potential helps to describe both the apparent charge of a nanoparticle, as well as the stability of a colloidal suspension. As the apparent charge of a suspended particle, the zeta potential is deeply related to the presence of ions in the surrounding media and to its pH. Additionally, it allows us to understand the interactions of the particles with themselves and with other nano molecules that may lead to flocculation. Thus, the zeta potentials of **@AcLi** and **THPP@AcLi** were measured in PB pH 7 (−23.42 ± 2.17 and −17.00 ± 1.67, respectively). A negative charge was observed for both nanoparticles, and no significant difference was found between them (two-way ANOVA, Sidak’s multiple comparison test, *P* > 0.05). As the values obtained for both nanoparticles were similar, it could be assumed that **THPP** did not exert an effect on the charge of the nanoparticle and in its zeta potential. Thus, for further analysis, nanoparticle zeta potential was measured at different pH values in 0.1 M buffers (Figure 5). Interestingly, both types of nanoparticles had a similar behavior, with an increase on the zeta potential value at pH 2, reaching a plateau and then decreasing at basic pH 9 and 10. However, we could observe that the addition of **THPP** into **@AcLi** nanoparticles leads to an increase on the zeta potential (Two-way ANOVA, Sidak’s multiple comparison test, *P* < 0.05), excepting pH 7 and pH 8 (two-way ANOVA, Sidak’s multiple comparison test, *P* > 0.05). The magnitude of the zeta potential can predict the stability of a colloidal suspension, being that suspensions with higher magnitudes of zeta potential tend to be more stable and less likely to flocculate (Kumar and Dixit, 2017). Thus, our findings indicate that at acidic pH, **@AcLi** are more likely to flocculate, reaching their highest stability in basic pH. As we found differences between **THPP@AcLi** and **@AcLi**, **THPP** exerts an effect in the apparent charge of the nanoparticles, increasing the likeness of nanoparticle flocculation, when compared with **@AcLi.**

To further demonstrate the viability of our process, we tested the stability of **THPP@AcLi** by monitoring the UV-vis absorption spectra of the nanoparticles suspended in PB pH 7, over 60 days, when stored in the dark at 25°C (Supplementary Material 4). **THPP@AcLi** demonstrated a high stability, with negligible **THPP** leaking, after 60 days of storage (9%). Additionally, the UV-vis profile did not change over time. Thus, **THPP@AcLi** withstood suspension in aqueous media in the dark and at 25°C, which makes it suitable for storage, without specific conditions in order to preserve the stability of the formulation.

### Acetylated Lignin Nanoparticles Singlet Oxygen Production

**FIGURE 6:**
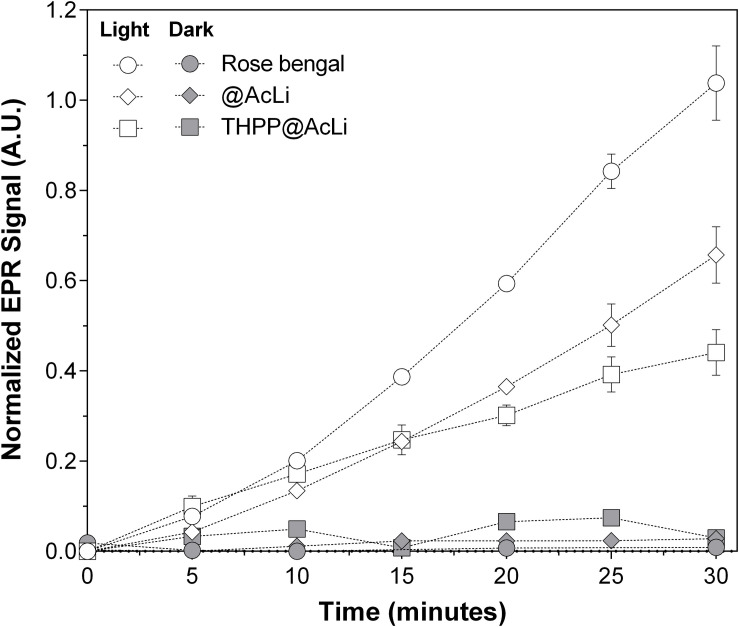
Monitoring of singlet oxygen production, as 2,2,6,6-tetramethylpiperidine-1-oxyl (TEMPO) radical detection through electron paramagnetic resonance (EPR) of acetylated lignin nanoparticles **(@AcLi)** [2 mg/ml] (diamonds) and 5,10,15,20-tetrakis(4-hydroxyphenyl)- 21*H*,23*H-*porphyrin-loaded @AcLi **(THPP@AcLi)** [**THPP** 60 μM, **AcLi** 100 μg/ml] (squares). Rose bengal [1.5 μM] (circles) was used as a reference. Singlet oxygen production was measured as a function of time under light irradiation (white symbols) or dark incubation (gray symbols).

It has been demonstrated that AcLi can produce ROS, specifically, singlet oxygen and superoxide anion (Marchand et al., 2018). We extrapolate these observations to the particular case of **@AcLi**, acting as photosensitizers in aqueous media and producing ROS. The singlet oxygen generated by **@AcLi** and **THPP@AcLi** was monitored by **TEMP** quenching, which easily reacts with singlet oxygen to form 2,2,6,6-tetramethylpiperidine-1-oxyl (**TEMPO**), a stable radical that can be detected with EPR spectroscopy (Riou et al., 2014; Marchand et al., 2018). In Figure 6, the nanoparticle suspensions in PB pH 7.4 of **@AcLi** (2 mg/ml) and **TPPOH@AcLi** (100 μg/ml lignin, 60 μM **THPP**) were compared, with or without light irradiation [halogen lamp, 270 μE/(s m^2^)]. Rose bengal (1.5 μM, DMF), a well-known photosensitizer and singlet oxygen generator, was used as a reference. It is worth mentioning that the concentration of **@AcLi** nanoparticles was 20 times superior to that of **THPP@AcLi** but showed similar singlet oxygen generation, meaning that **THPP@AcLi** is approximately 20 times more efficient at producing singlet oxygen under the tested conditions. Both **@AcLi** and **THPP@AcLi** singlet oxygen was light-driven, corroborated by the differences observed between the dark and light irradiated samples, after 30 min (two-way ANOVA, Sidak’s multiple comparison test, *P* < 0.01). The more efficient generation of singlet oxygen of **THPP@AcLi**, compared with **@AcLi**, demonstrated that **THPP** keeps its photosensitizing activity after encapsulation. Remarkably, the singlet oxygen produced by the encapsulated **THPP** is able to diffuse outside the nanoparticles, react with TEMP, and form the more stable TEMPO radical.

### Acetylated Lignin Nanoparticle Fluorescence

**FIGURE 7:**
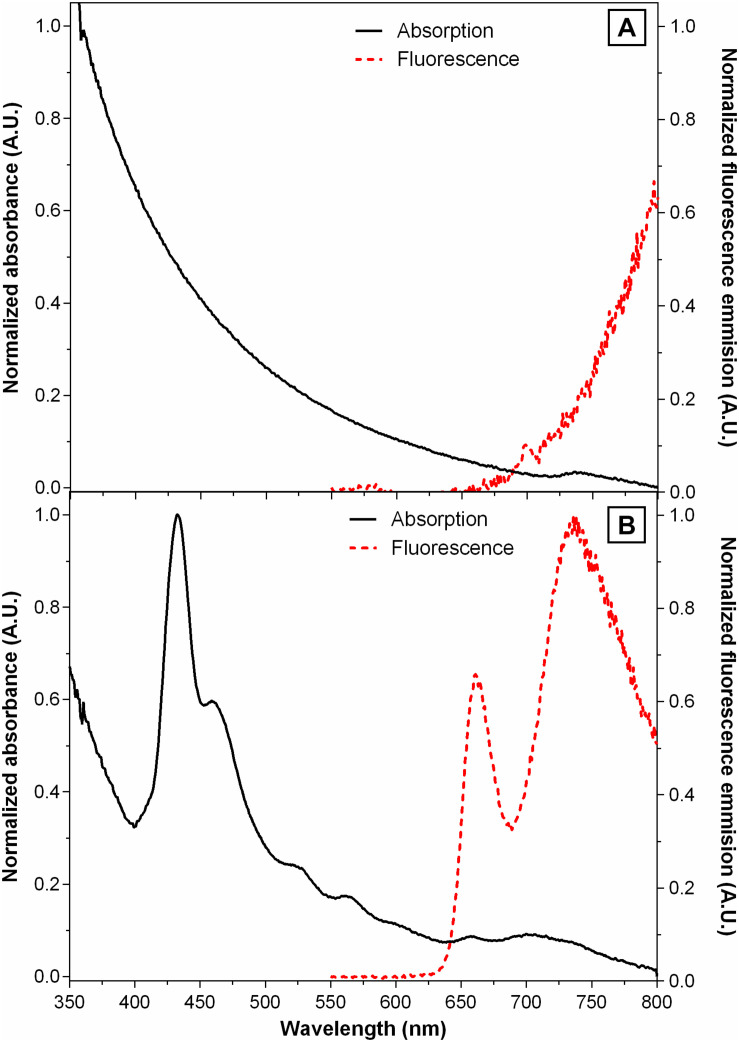
Normalized UV-vis absorption spectra (black lines) and fluorescence emission spectra (red lines) of **(A)** acetylated lignin nanoparticles **(@AcLi)** [30 μg/ml] and **(B)** 5,10,15,20-tetrakis(4-hydroxyphenyl)-21*H*,23 *H-*porphyrin-loaded **@AcLi (THPP@AcLi)** [**THPP** 2.5 μM, **AcLi** 30 μg/ml]; recorded in PB 0.1 M pH 7, room temperature, λ_*ex*_ = 425 nm.

It has been previously documented that molecular aggregation due to water coordination is a fluorescence quencher for porphyrins (Zannotti et al., 2018), a quantum phenomenon that is competitive with singlet oxygen production. To evaluate the extent of this issue, the fluorescent emission spectra of **@AcLi** (30 μg/ml) and **THPP@AcLi** (**THPP** 2.5 μM, **AcLi** 30 μg/ml) were measured as suspended in PB pH 7, with excitation at 425 nm (Figure 7).

Previous reports indicate that different chemical derivatives of lignin are fluorescent (Donaldson and Radotic, 2013). However, most of these observations have been done in organic solvents, where lignin is deployed without aggregation. Reports in the literature indicate that the fluorescence of lignin depends on the degree of aggregation, as the architecture of the nano-objects affects the interaction of the fluorophores (Xue et al., 2020). However, most of the studies on lignin fluorescence have been done with excitation wavelengths on the UV region, where lignin was known to strongly absorb. In our studies, we aimed at exciting the porphyrin, thus using excitation wavelength in the 400–500 nm range, where lignin did not absorb significantly. Thus, it is not surprising that a defined fluorescence band was not found for **@AcLi**; therefore, AcLi contribution to the fluorescence of **THPP@AcLi** was negligible in the tested conditions.

The **THPP@AcLi** emission spectrum, after excitation at 425 nm, showed two main peaks, a defined peak at 663 nm and a stronger less defined peak at around 733 nm. Additional experiments (Supplementary Material 5) showed that the peak found at 663 nm corresponds to **THPP** emission, while the peak at 733 nm could correspond to both **THPP** and **THPPH_2_^2+^** centered emission, as evidenced by the corresponding excitation spectra. The calculated quantum yield (Φ_*F*_) for **THPP@AcLi** is 0.0016 ± 0.0001, a value that is lower than the one reported for **THPP** (Φ_*F*_ = 0.17, DMF) (Ormond and Freeman, 2013). Nevertheless, the obtained fluorescence still represents a success, as previous experiments have demonstrated that the fluorescence of **THPP** in aqueous media (Φ_*F*_ = 0.00071, PB pH 7, with 2.5% DMSO) is almost completely quenched. Thus, the encapsulation of **THPP@AcLi** partially prevented the quenching of **THPP** in aqueous medium and additionally avoided the usage of organic solvents to increase the availability of **THPP**.

**FIGURE 8:**
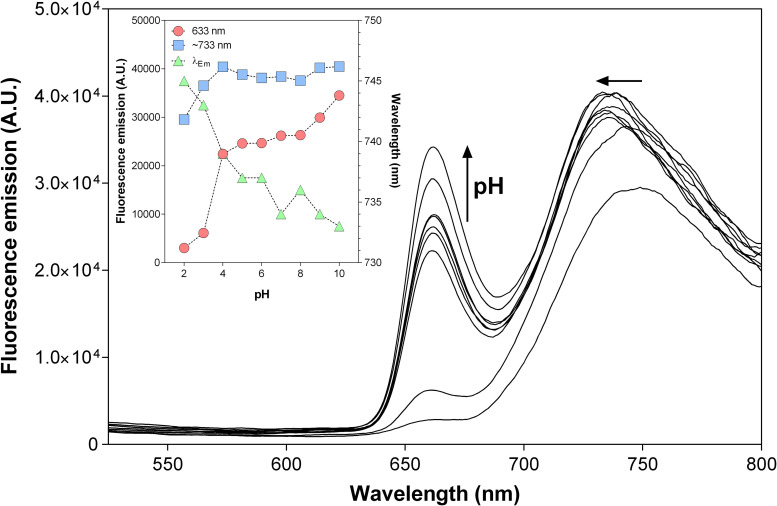
Emission spectra of 5,10,15,20-tetrakis(4-hydroxyphenyl)-21*H*,23*H-*porphyrin-loaded acetylated lignin nanoparticles **(THPP@AcLi)** [3 μM], as a function of pH [0.1 M glycine-HCl buffer (pH 2 and pH 3), 0.1 M acetate buffer (pH 4 and pH 5), 0.1 M phosphate buffer (0.1 M pH 6, pH 7, and pH 8), 0.1 M glycine-NaOH buffer (pH 9 and pH 10)]; recorded at room temperature, λ_*Ex*_ = 425 nm. Inset: evolution of the fluorescence intensities recorded at 663 nm (red circles), the maximum emission at around 733 (blue squares), and the wavelength for the maximum emission found for the ∼733 nm band (green triangles) as a function of pH.

Porphyrin’s fluorescence is sensitive to the medium, especially to the pH (Zannotti et al., 2018; Leroy-Lhez et al., 2019). Previously in this work, we have demonstrated that **THPP@AcLi** were resistant to the fluctuation of pH in the media, according to UV-vis absorption and zeta potential studies. To further corroborate our findings, the fluorescence emission of **THPP@AcLi** was also recorded at different pH values (Figure 8). We could observe a pronounced decrease of fluorescence intensity with pH at 663 nm (Figure 8, inset), while the intensity of the fluorescence of the second band remained stable. Concomitantly, a red-shift of the wavelength of emission for this second band was also observed (from 733 nm at pH 10 to 745 nm at pH 2). This was not surprising as the peak at 663 nm is related to **THPP**, which in acidic media transforms into the protonated species **THPPH_2_^2+^**; meanwhile, the second peak was related to both species and was thus affected by the equilibrium between **THPP** and **THPPH_2_^2+^** as a function of pH. Therefore, at a low pH value, **THPPH_2_^2+^** must be the predominant species, characterized by an emission at higher wavelength than **THPP**. However, when Φ_*F*_ was calculated for the whole pH range, it was found that besides variations on the emission profile, Φ_*F*_ remained stable (∼0.16) (Supplementary Material 6). This is consistent with our previous results, where we observed changes on the absorption spectra at acidic conditions. Nevertheless, the global quantum yield remained stable at different pHs.

### Photodynamic Antimicrobial Chemotherapy Effect of Porphyrin-Loaded Nanoparticles Against Bacteria

The nanoparticles were tested against five bacterial strains, three Gram-positive (*S. aureus*, *S. epidermidis*, and *E. faecalis*) and two Gram-negative (*E. coli* and *P. aeruginosa*). The highest concentration of **THPP** encapsulated in **@AcLi** was 50 μM, corresponding to 0.33 mg/ml of **AcLi.** For **@AcLi**, the highest concentration used was 1.6 mg/ml; reports in the literature indicate that at this concentration, lignin nanoparticles were innocuous to human cells (Alqahtani et al., 2019). The results found in this study showed that **@AcLi** have a bacteriostatic effect at 1.6 mg/ml; nevertheless, **@AcLi** do not have a bactericidal effect at the highest concentration tested (Supplementary Material 7).

**FIGURE 9:**
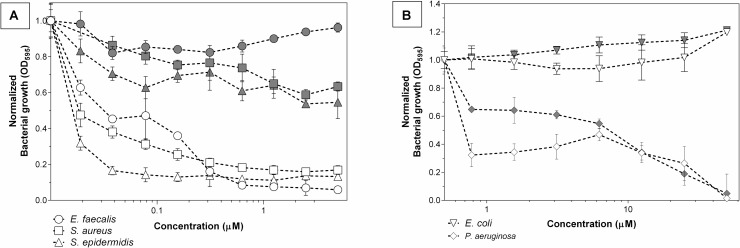
Bacteriostatic effect of 5,10,15,20-tetrakis(4-hydroxyphenyl)-21*H*,23*H-*porphyrin-loaded acetylated lignin nanoparticles **(THPP@AcLi)** after light irradiation (white LED light dose, 4.16 J/cm^2^, white symbols) or dark incubation (gray symbols) against **(A)** three Gram-positive bacteria and **(B)** two Gram-negative bacteria.

First, **THPP@AcLi** were evaluated as bacteriostatic agents, analyzing its capability to arrest bacterial growth. **THPP@AcLi** demonstrated a high capacity to diminish the growth of Gram-positive bacteria, after 1 h of irradiation, under a white LED light dose (4.16 J/cm^2^). For Gram-positive inactivation, concentrations as low as 0.078 μM were enough to diminish growth at around 85% (Figure 9A). On the other hand, **THPP@AcLi** was not able to diminish the growth of Gram-negative *E. coli* but seemed to exert a bacteriostatic non-photodynamic effect on *P. aeruginosa* (Figure 9B). The most sensitive strain was *S. epidermidis*, followed by *E. faecalis* and, lastly, *S. aureus*. Usually, in PACT, low dark toxicities are desired, as it ensures that the antimicrobial effect is only triggered by light irradiation. Our results fulfill this necessity, as when using 0.640 μM of **THPP@AcLi**, the bacterial growth of *E. faecalis* was less than 10% after light irradiation; meanwhile, at dark incubation, the bacterial growth in the dark was around 85%. Similar results were found with *S. aureus* and *S. epidermidis* (Supplementary Material 8).

Although it can be addressed that growth arrest was due to the cellular death, it can also be provoked by a decrease on the bacterial metabolism or due to cellular damage, which may be overcome with enough recovery time. The difference between bacteriostatic and bactericidal effect is dose-dependent. Thus, the bacterial survival was assessed under similar conditions. As previously observed, **THPP@AcLi** was not effective against Gram-negative bacteria (Supplementary Material 8). Indeed, **THPP@AcLi** was unable to diminish the Gram-negative bacterial survival rate at 50 μM either at light (white LED light dose, 4.16 J/cm^2^) or dark conditions (two-way ANOVA, Sidak’s multiple comparisons test, *P* > 0.05).

**FIGURE 10:**
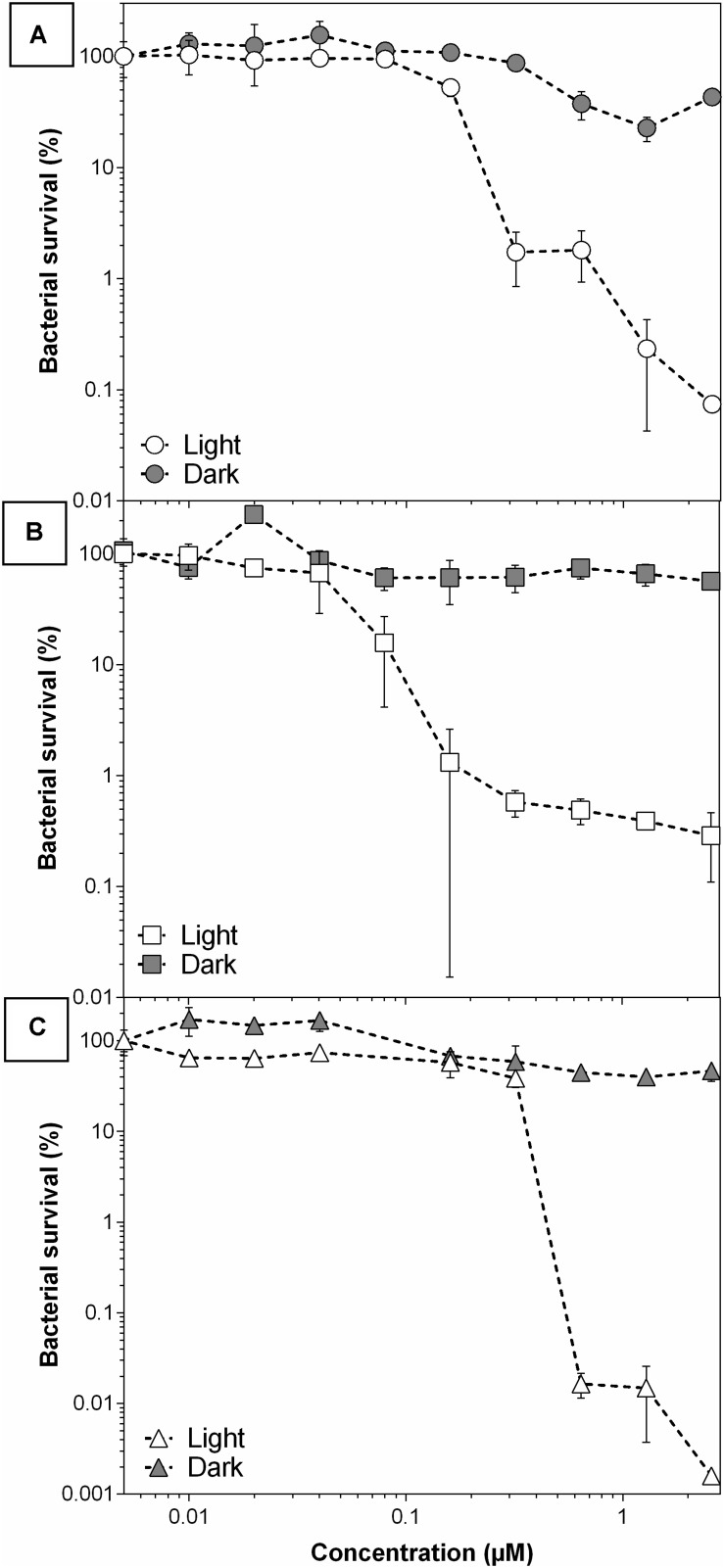
10 | Bacterial survival of **(A)** *S. aureus*, **(B)** *S. epidermidis*, and **(C)** *E. faecalis*, with 5,10,15,20-tetrakis(4-hydroxyphenyl)-21*H*,23*H-* porphyrin-loaded acetylated lignin nanoparticle **(THPP@AcLi)** treatment at different concentrations after light dose (4.16 J/cm^2^) or dark incubation.

Otherwise, several concentrations were tested for **THPP@AcLi** against the Gram-positive strains. They demonstrated a great efficiency at killing bacteria, being able to destroy up to 99.9999% of *E. faecalis* (Figure 10). Previous experiments (Figure 9) demonstrated a low dark chemotoxic effect for **THPP@AcLi**; in agreement, similar results were found on the bacterial survival rate (Supplementary Material 10), with differences between the light and dark conditions of several orders of magnitude. We found an efficient bactericidal effect at concentrations as low as 2.5 μM of **THPP@AcLi** and just 4.16 J/cm^2^ of white LED light dose.

Our previous experiments had demonstrated that **THPP@AcLi** were stable at a wide range of pH. This is important for antibacterial applications. Usually, bacteria are viable within a limited range of pH values; with pathogenic bacteria being viable at a range between 5.5 and 8 (Madigan et al., 2014). However, bacterial metabolism provokes changes in the pH in different ways. Excretion of organic acids, such as propionic acid and isopropylic acid, can decrease the pH of the surrounding media, while amine compounds, formed through the degradation of amino acids and proteins, can increase the pH of the bacterial surrounding media (MacFaddin, 2000). Additionally, the pH of the medium has been found to influence the efficiency of several antibiotics (Yang et al., 2014). PACT is usually addressed as a topical treatment or for surface disinfection due to the difficulty of irradiating the inside of a living being (Wainwright et al., 2017). Thus, a formulation that works on a wide range of pH is desirable, as it can withstand the changes provoked by bacteria or the conditions found on several surfaces.

**FIGURE 11:**
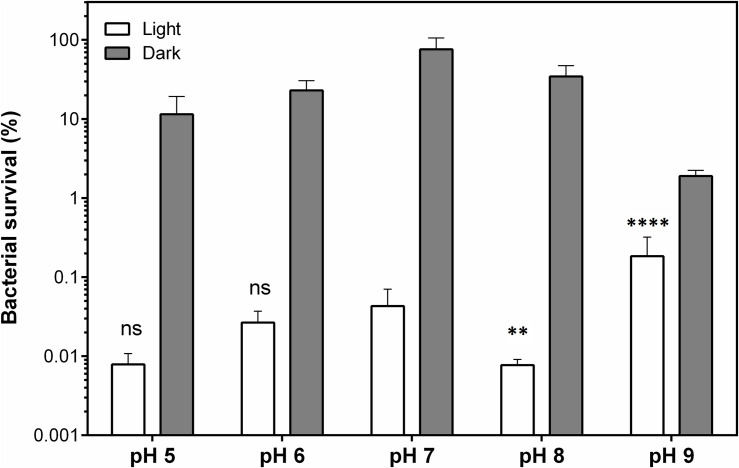
Bacterial survival of *S. aureus*, as a function of pH, when treated with 5,10,15,20-tetrakis(4-hydroxyphenyl)-21*H*,23*H-*porphyrin-loaded acetylated lignin nanoparticles **(THPP@AcLi)** 2.5 μM under light irradiation (white LED light dose, 4.16 J/cm^2^) or dark incubation. A two-way ANOVA analysis was made, with a Sidak’s multiple comparison test, to analyze the differences found between pH 7 and the other pH conditions tested (ns, non-significant *P* > 0.05; ^∗∗^*P* < 0.01; ^****^P < 0.0001). At all pH conditions, a difference was found between the light and dark conditions (*P* < 0.0001).

In that perspective, **THPP@AcLi** was tested against *S. aureus* on aqueous media from pH 5 to pH 9, at a concentration of 2.5 μM, where we had previously observed a decrease of bacterial survival of at least 99.9% (Figure 11). Other pH conditions were tested, but as the bacterial controls demonstrated being unviable, these results were not included in the analysis. **THPP@AcLi** was able to diminish bacterial survival at all the pH conditions, through an effective photodynamic effect observed, with differences between light irradiation and dark incubation samples (two-way ANOVA, Sidak’s multiple comparisons test, *P* < 0.0001). When compared with the PACT effect obtained at pH 7, no differences were found at pH 5 and pH 6 (*P* > 0.05). Nevertheless, at pH 8, the PACT effect had a slight improvement (*P* < 0.01), while at pH 9, the bacterial survival increased up to 0.184 (*P* < 0.0001). Although statistically there were some differences found, in general, the PACT effect permitted a bacterial survival below 0.2%. The fluctuation of the values found could be attributed to either the pH effect on the cells or the buffer composition, as three buffers with different compositions were used for this experiment. Thus, **THPP@AcLi** PACT effect was stable at a wide range of pH. Their stability corresponded to our previous observations, where their photophysical characteristics remained relatively stable at different pH conditions. This good correlation between the photophysical properties and their biological applications enhanced the applications spectra for **@AcLi** loaded with a photosensitizer.

**FIGURE 12:**
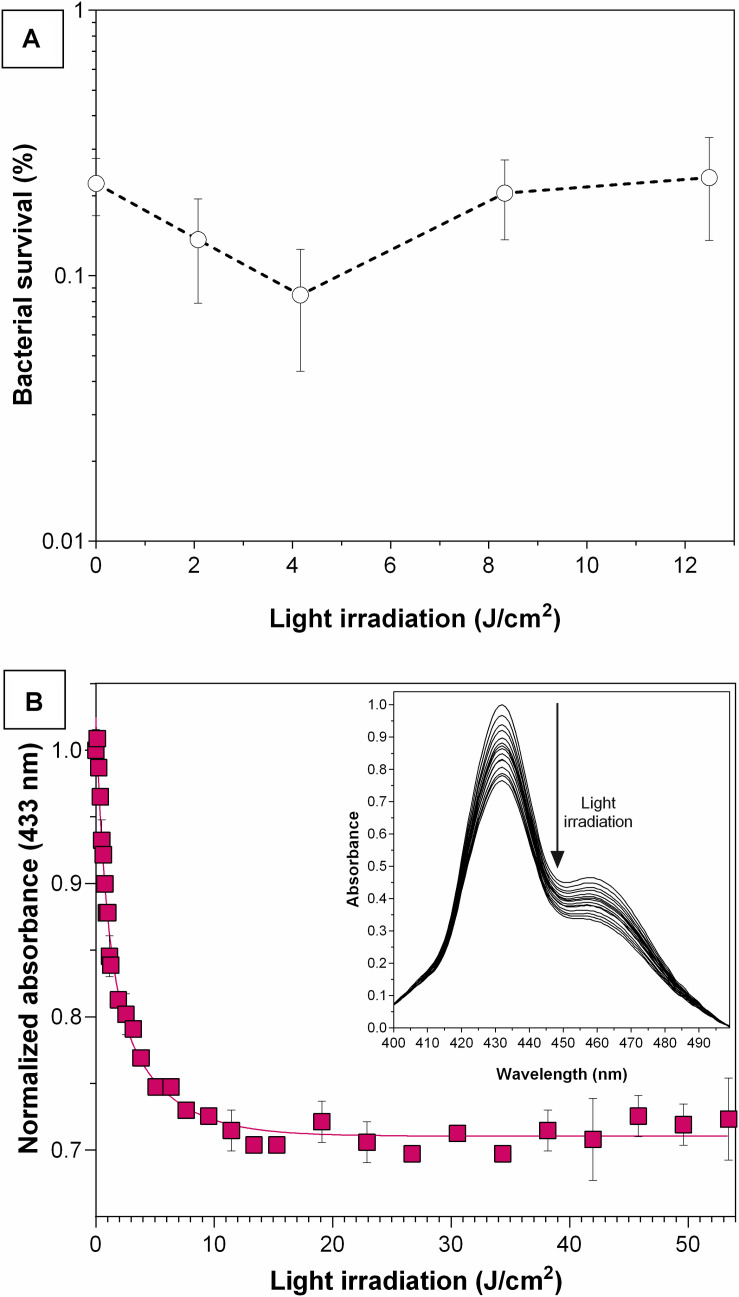
Effect on 5,10,15,20-tetrakis(4-hydroxyphenyl)-21 *H*,23*H-*porphyrin-loaded acetylated lignin nanoparticles **(THPP@AcLi)** of previous light irradiation. **(A)** Photoeradication of *S. aureus* (white LED light dose, 4.16 J/cm^2^), with nanoparticles previously irradiated with white LED light. As a control, a non-irradiated sample was used (0 J/cm^2^). **(B) THPP@AcLi** absorbance at 433 nm was monitored through its irradiation with white LED light (1 mW/cm^2^). Inset: the spectra between 400 and 500 nm are observed for the whole irradiation time.

In order to further demonstrate the stability of **THPP@AcLi**, their resistance to light irradiation was assessed. **THPP@AcLi** were exposed to light irradiation periods before incubation with bacteria under conditions similar to previously done PACT experiments. Afterward, the irradiated nanoparticles were mixed with *S. aureus* bacteria and the PACT irradiation was carried out, as routinely for bacterial eradication. A non-irradiated control was used, and bacterial survival was reported after *S. aureus* photodynamic eradication (Figure 12A). The **THPP@AcLi** withstood the light irradiation, remaining as effective as the previously non-irradiated sample (0 J/cm^2^). This would allow nanoparticles to remain functional after long irradiation periods or after several cycles of usage. To analyze the effect of light irradiation on **THPP@AcLi**, nanoparticles were irradiated under similar conditions and the absorbance at 433 nm was monitored after irradiation in order to evaluate potential degradation due to light-driven self-annihilation (Figure 12B). Interestingly, the absorption of the Soret band diminished quickly and then reached a plateau, with around 73.32% of the original absorbance found, even after 7 h of constant irradiation (1 mW/cm^2^). By analyzing the UV-vis absorption spectra, a decrease on the B-band intensity at 457 nm was also observed, with changes in the ratio between this and the Soret band (initial A_437_/A_452_ 0.465, final A_437_/A_452_ 0.334). As the B-band can be attributed to the protonated molecule **THPPH_2_^2+^** according to the literature (Zannotti et al., 2018; Leroy-Lhez et al., 2019), we can conclude that this species was more sensitive to photobleaching than the non-protonated one.

### 5,10,15,20-Tetrakis(4-Hydroxyphenyl)-21H,23H-Porphyrin Inside Acetylated Lignin Nanoparticle Interaction With Bacteria

**FIGURE 13:**
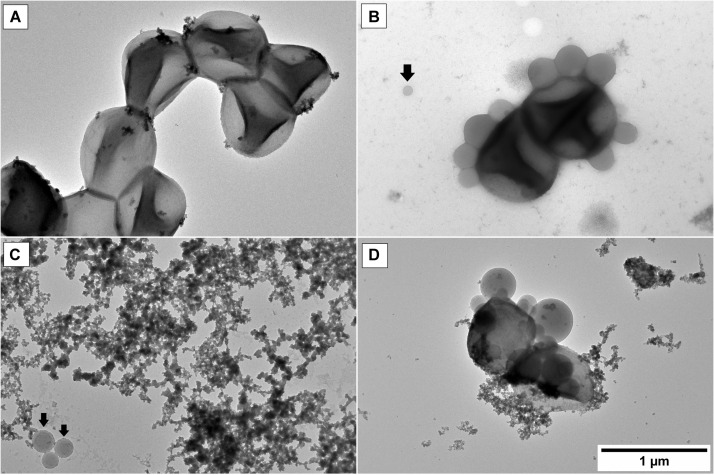
Transmission electron microscopy (TEM) observations of **(A)** *S. aureus* cells *in vivo*; **(B)** *S. aureus* and 5,10,15,20-tetrakis(4-hydroxyphenyl)-21*H*,23*H-* porphyrin-loaded acetylated lignin nanoparticles **(THPP@AcLi)**, the black arrow indicates a non-bound nanoparticle; **(C)** and **(D)** *S. aureus* and **THPP@AcLi** after light irradiation (incandescent bulb, 2,500 luxes, 5 min).

In this work, the stability of **THPP@AcLi** photophysical properties has been demonstrated, being effective against Gram-positive bacteria at different pH values, even after previous light irradiation. However, the previous experiments had not clarified how bacteria are eradicated, as is less likely that the photosensitizer gets in direct contact with bacteria. The uptake of porphyrins and other photosensitizers by bacteria had been widely studied (Ferro et al., 2007; Orekhov et al., 2018), but our results suggested that **THPP@AcLi** do not leak out the photosensitizer. In order to have an insight into the interaction between bacteria and **THPP@AcLi**, we made TEM observations over a mixture of bacteria and nanoparticles. *S. aureus* cells were observed without typical chemical fixation (Figure 13A). The observed cells did not have the characteristic round shape found for staphylococci bacteria; rather, they had a “squashed” shape likely due to the acidic uranyl acetate fixation/staining. When nanoparticles were mixed, a spontaneous binding was observed, as no incubation time elapsed for the first observation (Figure 13B). Interestingly, the amount of observable free nanoparticles was low when compared to the observed bound nanoparticles. Nanoparticles were observed surrounding the surface of bacteria. The attached nanoparticles were observed as spheres, with only a partial merge within the bacterial membrane. This suggested that nanoparticles do not penetrate inside the cell but remained in the outskirts of the membrane. Preparations made from a mixture of bacteria and nanoparticles, done 30 min ahead, still presented this pattern. Thus, the penetration of nanoparticles inside bacteria is not time dependent. The mixture of nanoparticles and bacteria was irradiated *in situ* over the copper grid with an incandescent bulb light (2,500 lux, 5 min) and was observed at TEM. After light irradiation, bacteria were scarce and cellular debris was observed throughout the place. Bacteria were also found surrounded by **THPP@AcLi** (Figures 13C,D). In some cases, bacteria were observed while spilling their cellular contents (Figure 13D). The cellular contents include proteins and nucleic acids, which are transparent to the TEM, but we were able to observe them after being contrasted with uranyl acetate, observable at TEM, as black debris. The TEM observations suggest that bacteria suffered extensive damage on their cellular wall when exposed to light and **THPP@AcLi**. As presumably nanoparticles were unable to completely penetrate inside the cell, a local ROS production against the cellular wall was likely to trigger the photodynamic effect. Insufficient damage to the bacterial wall may provoke an arrest on the bacterial growth, which corresponds to our previous observations of a large bacteriostatic effect for **THPP@AcLi**.

An interesting observation was the spontaneous binding of THPP@AcLi with bacteria. It had been previously observed that lignin nanoparticles worked as flocculants, capturing *E. coli* and *S. aureus* (Yin et al., 2018). This effect was observed in our experiments, where the mixture of bacteria and nanoparticles quickly flocculated to the bottom of the flask. The high affinity of bacteria and THPP@AcLi may work as a synergic system. Although we had observed through EPR a low production of singlet oxygen for THPP@AcLi, the tight interaction between bacteria and nanoparticles results in a higher concentration of ROS at the cellular level. Thus, both flocculant and photodynamic effect could be a promising alternative as a PACT system. Such system could find wide applications in wastewater purification, a complex media with biological and chemical pollutants that require disinfection before release into the environment. Purification of wastewater usually comprises physical methods, involving sedimentation, aeration, and filtration, and at these steps, lignin could be used as an alternative.

## Discussion

Our results demonstrate that **THPP** encapsulation into @AcLi is an easy, effective, and reproducible method, increasing the value of lignin as a biopolymer with biomedical applications. Besides, this encapsulation method may be suitable for a wide range of molecules, which are our current subject of study.

The effect of aggregation on porphyrins and tetrapyrrolic compounds has been addressed as one of the main issues for biological applications of both PDT and PACT, as aggregation quenches ROS production, diminishing their efficiency and potential (Liu et al., 2018). The present work has produced a formulation that efficiently delivers **THPP** into an aqueous media, with less aggregation than the non-encapsulated **THPP**. Besides, **THPP** inside **@AcLi** is able to withstand a wide pH range without being affected, as demonstrated by its UV-vis absorption and fluorescence emission properties. Additionally, the pH stability is extended to its physicochemical properties, as its zeta potential, and the changes in the pH of the media do not affect their efficiency against bacteria.

Lignin nanoparticles are currently under research as vehicles for small molecules, with reports of nanoparticles being able to diffuse the small molecules over time or over pH changes (Zhou et al., 2019). Nevertheless, we were able to demonstrate that **THPP@AcLi** are stable over time, releasing less than 10% of **THPP** into the surrounding media after 60 days of observations. However, the stable encapsulation of **THPP** inside nanoparticles leads to the question if ROS generated by **THPP** would be able to escape from the nanoparticles and actually produce an observable macroscopic effect. Through EPR, we detected singlet oxygen generation for **@AcLi** and for **THPP@AcLi**. However, the evidence has demonstrated that **THPP@AcLi** singlet oxygen generation is mostly due to **THPP**, as **THPP@AcLi** is 20 times more efficient at producing singlet oxygen than **@AcLi**. Then, singlet oxygen generated by **THPP** was able to diffuse through the**@AcLi**.

Although it has been indicated that a light dose of 4.16 J/cm^2^ is necessary to kill bacteria, recent reports have indicated that the UV-vis absorbance of PACT and PDT molecules need to be taken into account for a corrected light dose (Schaberle, 2018). With this correction done (Supplementary Material 2), **THPP@AcLi** is only able to absorb 65% of the white LED light irradiated on it, resulting in a corrected light dose of 2.71 J/cm^2^. Our experiments had demonstrated that the nanoparticles were stable under light doses 20 times higher than the corrected light dose, and thus higher doses could be applied on bacteria, while maintaining their efficiency.

In regard to bacterial eradication, **THPP@AcLi** was only able to diminish the bacterial growth and survival of Gram-positive bacteria. This is not surprising, as PACT has been described as more effective against Gram-positive bacteria than against Gram-negative ones (Huang et al., 2012). The differences in the efficiency were addressed to be due to the impermeability of its double membrane, a common problem with the development of successful antibiotic treatments (Nikaido, 2003). Interestingly, PACT applications on Gram-negative bacteria had overcome this obstacle through the usage of cationic photosensitizers (Ragàs et al., 2010; Cieplik et al., 2018; Aroso et al., 2019) or photosensitizers linked to antimicrobial peptides (Le Guern et al., 2017, 2018), which have a high affinity for the anionic heads of the lipopolysaccharides, facilitating the interaction between bacteria and photosensitizer. Interestingly, in our work, we observed that although nanoparticles have a negative charge, they seem to strongly interact with bacteria, demonstrated by TEM observations. Thus, further investigations are underway with cationic molecules inside **@AcLi**, aiming for Gram-negative bacteria eradication. Additionally, lignin modifications could lead to the construction of cationic lignin nanoparticles. Nevertheless, we had observed strong spontaneous interactions between nanoparticles and bacteria. It has been previously addressed that lignin nanoparticles were prone to act as flocculant agents, working as a physical method for water disinfection (Yin et al., 2018). Our strategy combines physical decontamination and a light-driven chemotoxic effect, a combination that could be ideal for wastewater decontamination. Wastewater is a complex mixture that needs to be purified before being released into the environment. Acetylated lignin nanoparticles could be used for light-driven water purification and, at the same time, for physical removal of bacteria and bacterial debris.

This work represents a cornerstone on lignin applications, as it is the first time it has been used on PACT applications. The present work, although able to eradicate Gram-positive bacteria, was unable to affect the survival of Gram-negative bacteria. Our future work comprises the modification of lignin with “sticky” moieties and the encapsulation of cationic porphyrins, hoping to obtain a wide-range formulation for antibacterial and antibiofilm purposes. Additionally, our future work aims to enhance the comprehension of the mechanism of the interaction between THPP/AcLi/bacteria through porphyrin uptake experiments and flow cytometry.

## Conclusion

Acetylated lignin nanoparticles were able to encapsulate a porphyrinic compound, **THPP**. Additionally, the encapsulation system was stable at a wide pH range, conserving its physical and photophysical properties, without leaking the encapsulated compound. Furthermore, it was demonstrated that this system was able to produce ROS and exert a photodynamic eradication effect on three Gram-positive strains. The photodynamic eradication could be due to a synergic effect of the flocculant properties of lignin nanoparticles and the light-driven ROS production of **THPP**. The encapsulation of further photosensitizers is likely to improve the presented results, and this strategy is currently under study for water decontamination and other applications.

## Data Availability Statement

The raw data supporting the conclusions of this article will be made available by the authors, without undue reservation, to any qualified researcher.

## Author Contributions

NM-C, GM, and NV participated on the preparation of the raw material and the nanoparticles. NM-C, GM, and SL-L participated on the characterization of the physico-chemical properties of the raw material and the nanoparticles. NM-C and CC measured the singlet oxygen production through EPR. NM-C and T-SO designed and performed the microbiological experiments. NM-C, KP, AŻ, and KM designed and performed the TEM observations. NM-C, MMP, and MC designed and performed the fluorescence spectroscopy experiments. All authors discussed the results and commented on the manuscript.

## Conflict of Interest

The authors declare that the research was conducted in the absence of any commercial or financial relationships that could be construed as a potential conflict of interest.
