# Supplementary material for: Porphyrin-Loaded Lignin Nanoparticles Against Bacteria: A Photodynamic Antimicrobial Chemotherapy Application
Source: Front Microbiol. 2020 Nov 17;11:606185. doi: 10.3389/fmicb.2020.606185 (PMC7705181; doi:10.3389/fmicb.2020.606185)
Supplement: Supplementary file 1 [file Presentation_1.pdf]

Supplementary Material

Supplementary Material

Table of Contents

1. Preparation of samples for transmission electron microscopy observations..................................1
2. Light Dose correction...2
3. Acetylated lignin characterization...3
4. Stability and leaking of THPP inside acetylated lignin nanoparticles 6
5. Excitation spectrum of THPP@AcLi...7
6. Fluorescent quantum yield of THPP@AcLi as a function of pH 9
7. PACT effect of @AcLi 10
8. Bacterial growth of five bacterial strains after THPP@AcLi PACT 13
9. Bacterial survival of three Gram positive strains after THPP@AcLi PACT...............................14
References 14

1. Preparation of samples for transmission electron microscopy observations

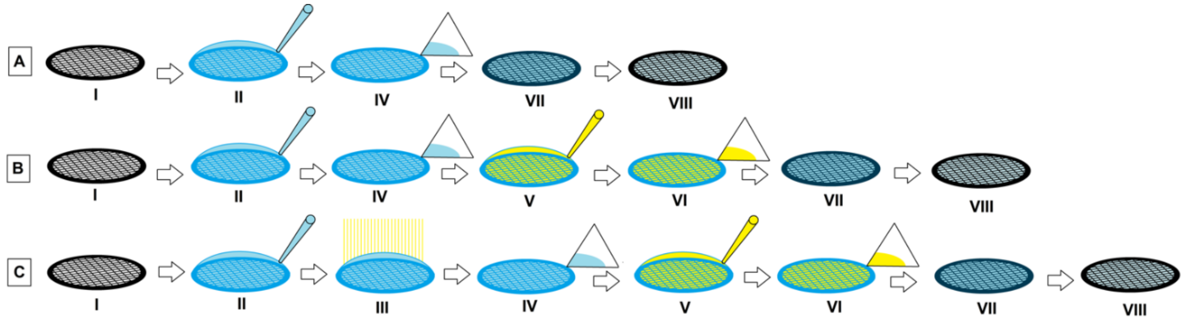

Figure S1. Transmission electron microscopy preparation process for nanoparticles (A), bacteria (B) and nanoparticle-bacteria interaction (C): I - clean and hydrophilic carbon grid, II - sample drop, III - light irradiation, IV - sample blot, V - negative stain drop, VI - negative stain blot after 60s, VII - air-drying, VIII - sample ready for observation.

2. Light Dose correction

Light dose correction was done, accordingly to the literature (Schaberle, 2018). For this, the power of the light emission source was obtained with a handheld power meter (LaserCheck, Coherent), and the light dose calculation was obtained using the equation S1:

$$LDC = \frac{\frac{\sum P(\nu)}{\nu}(1-10^{-A(\nu)})}{\frac{1}{\nu_r}(1-10^{-A_r})} \qquad \text{(Equation S1)}$$

with P(ν) being the emission of the light source at a certain wavenumber (v, cm^{-1}), A(ν) the absorption of **THPP** at a certain wavenumber, ν_r the wavenumber of the Soret band and A_r the absorbance of the Soret band. The obtained light dose correction, is the ratio of light that it is actually absorbed by the compound (Figure S2).

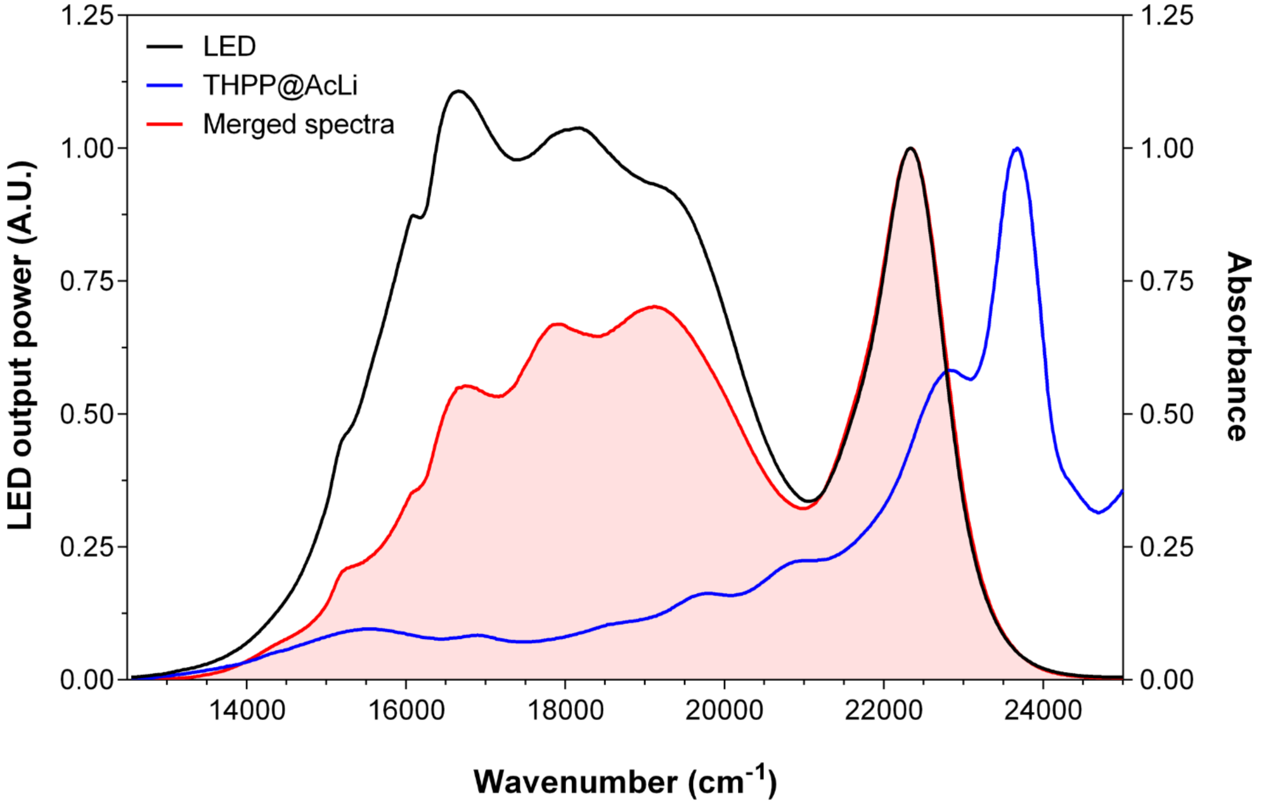

Figure S2. Normalized spectrum of the LED light output power (black line), the absorption spectrum of **THPP@AcLi** in PB pH 7 (blue line), and the merged spectrum (red line), with its area under the curve (light red) corresponding to the light dose correction rate.

3. Acetylated lignin characterization

Lignin acetylation and the photosensitive properties of acetylated lignins have been recently studied on another paper (Marchand *et al.*, 2018). Acetylated lignin (**AcLi**) was prepared as previously described and it was obtained as a crystalline brilliant brown powder. The material was characterized through FTIR (Figure S3.1).

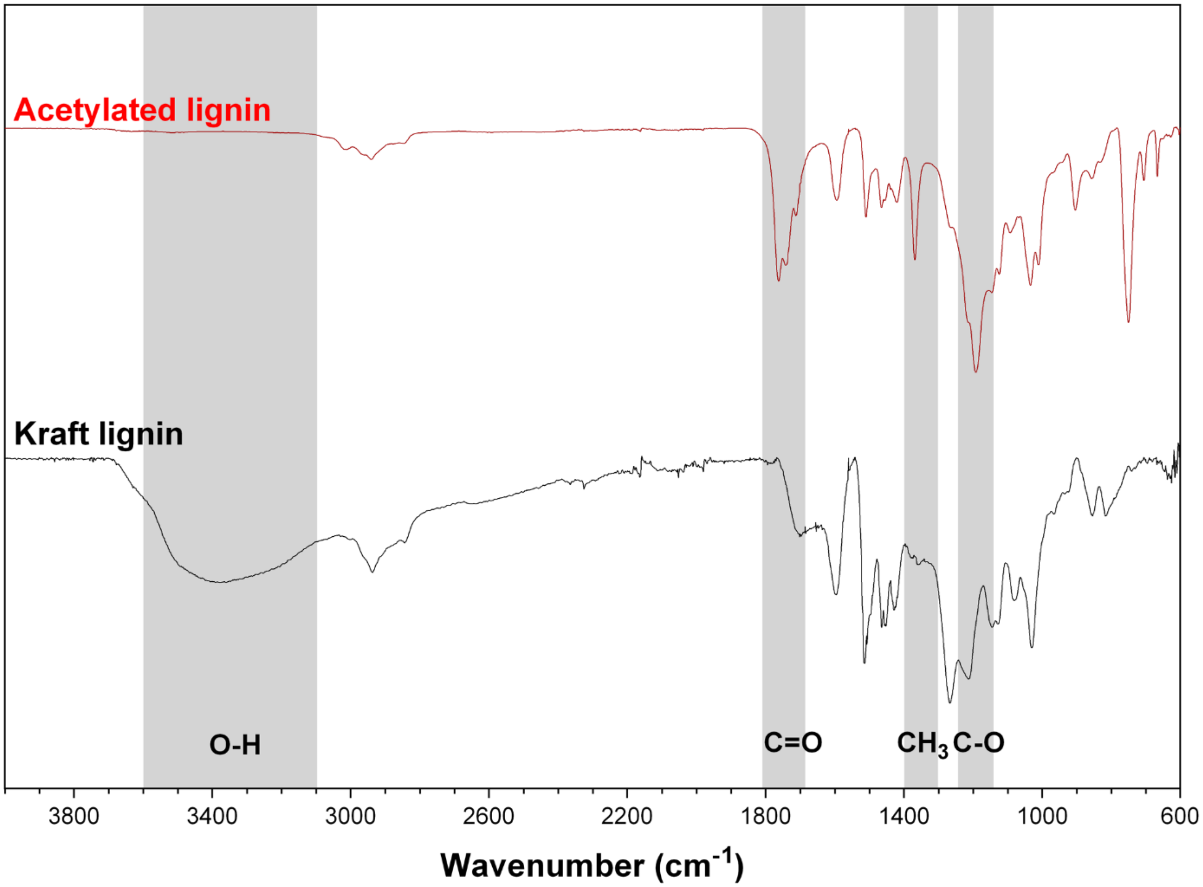

Figure S3.1. FTIR spectra of **AcLi** and raw kraft lignin. Significant absorbance patterns are gray highlighted.

The substitution of the hydroxyl groups at **AcLi** was observed through the disappearance of the wide O-H bond stretching band between 3100 and 3600 cm^{-1}, and with the appearance of a band at 1191 cm^{-1}, corresponding to the C-O ester bond stretch. Additionally, the lignin acetylation was confirmed through the appearance of the bands corresponding to C=O aromatic ester bond stretching and C=O aliphatic ester bond stretching, respectively at 1761 and 1739 cm^{-1}. The CH_3 moiety, corresponding to the acetyl group, appeared at 1464 cm^{-1}, as previously reported (Qian *et al.*, 2014; Marchand *et al.*, 2018).

The UV-vis absorption spectra of **AcLi** was recorded in acetonitrile at increasing concentrations, ranging from 0.010 to 0.400 mg/mL (Figure S3.2).

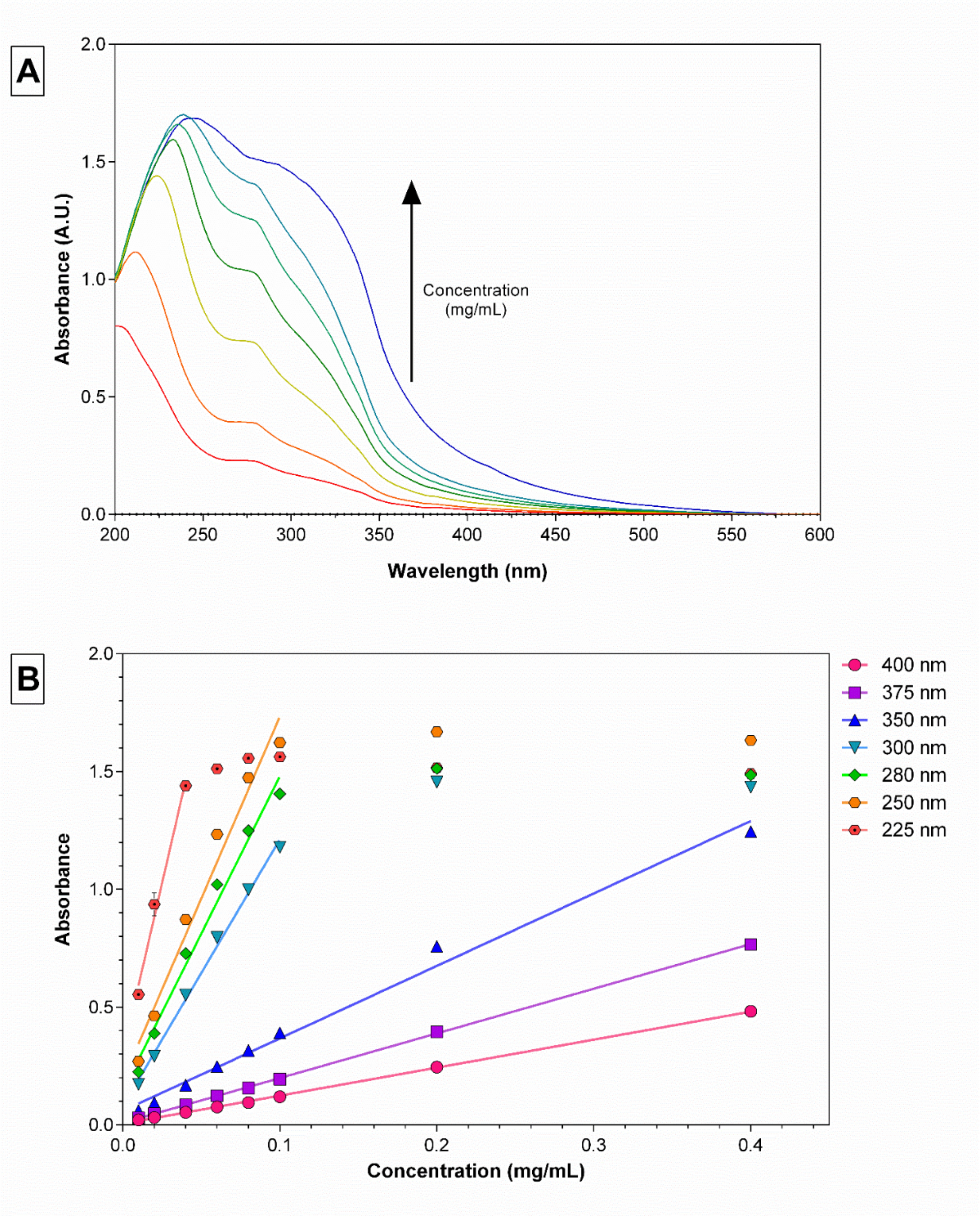

Figure S3.2. UV-vis characterization of **AcLi** in acetonitrile. (A) UV-vis spectra at increasing concentrations. (B) Absorbance of **AcLi** at several wavelengths, as a function of the concentration of the sample.

Interestingly, the **AcLi** UV-vis absorption profile is concentration sensitive; a defined band was observed at around 205 nm at 10 μg/mL, but at increasing concentrations, the band suffered a

progressive red shift from 205.5 to 248 nm, at 400 µg/mL, concomitantly with the appearance of a small peak at 280 and a shoulder at around 325 nm. The characteristics of **AcLi** absorption profile required the test of several random wavelengths to monitorize the changes of absorption as a function of concentration, and calculate its extinction coefficient (Table S3.1). The epsilon was calculated in a range where the increase demonstrated a linear behaviour, with a R square of at least 0.95. The best results were obtained on wavelengths above 350 nm, where the analytical range is bigger (0.01 – 0.4 mg/mL). Nevertheless, it would still be possible to use other wavelengths to determine analyse the **AcLi** concentration, on a smaller range of concentrations. For the present work, 350 nm was routinely monitored for determining the concentration of **AcLi** in nanoparticles samples.

Table S3.1. Epsilon calculated for **AcLi** at different wavelengths.

Wavelength (nm)	Range (mg/mL)	R Square (R^2)	ε (L/g cm)
400	0.01 – 0.4	0.9979	1.189 ± 0.01178
375	0.01 – 0.4	0.9986	1.897 ± 0.01527
350	0.01 – 0.4	0.9896	3.077 ± 0.06898
300	0.01 – 0.1	0.9946	11.33 ± 0.2160
280	0.01 – 0.1	0.9825	13.37 ± 0.4603
250	0.01 – 0.1	0.9724	15.44 ± 0.6713
225	0.01 – 0.03	0.9830	28.92 ± 1.440

4. Stability and leaking of THPP inside acetylated lignin nanoparticles

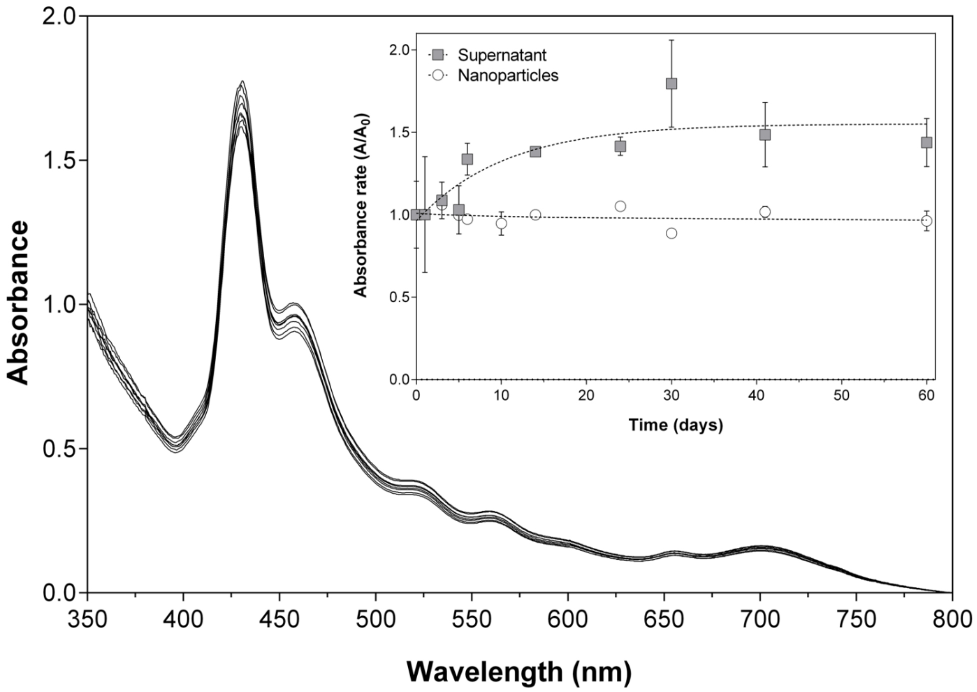

Figure S4. UV-vis absorbance spectra of the **THPP@AcLi** 2.39 μM in PB 0.1 M pH 7, followed up during 60 days. In the insert, the absorbance of the Soret band (430 nm) for the nanoparticles and the supernatants.

5. Excitation spectrum of THPP@AcLi

For further analysis, the excitation spectra recorded for observation wavelengths at 663, 733 and 780 nm were analyzed (Figure S5B). Interestingly, the excitation spectrum at 663 nm corresponds to the spectra of **THPP**, without presence of the protonated **THPP**H_2^{2+}. However, excitation spectrum at 733 and 780 nm showed two main peaks at 426 nm and 453 nm, which correspond to the Soret band and the B-band; the Q bands are observed at 521, 559, 595 and 652 nm for all the excitation spectra. In order to know if the porphyrinic species could be analyzed separately, the emission spectra were recorded at several excitation wavelengths (Figure S5C). Excitation at the B-band (455 nm) lead to a spectrum without the 663 nm band. However, excitation at other wavelengths always showed both emission bands, which is not surprising, as UV-vis absorption spectra of both species, **THPP** and **THPP**H_2^{2+} have spectral overlap in the whole range of wavelength, but between 450 – 475 nm.

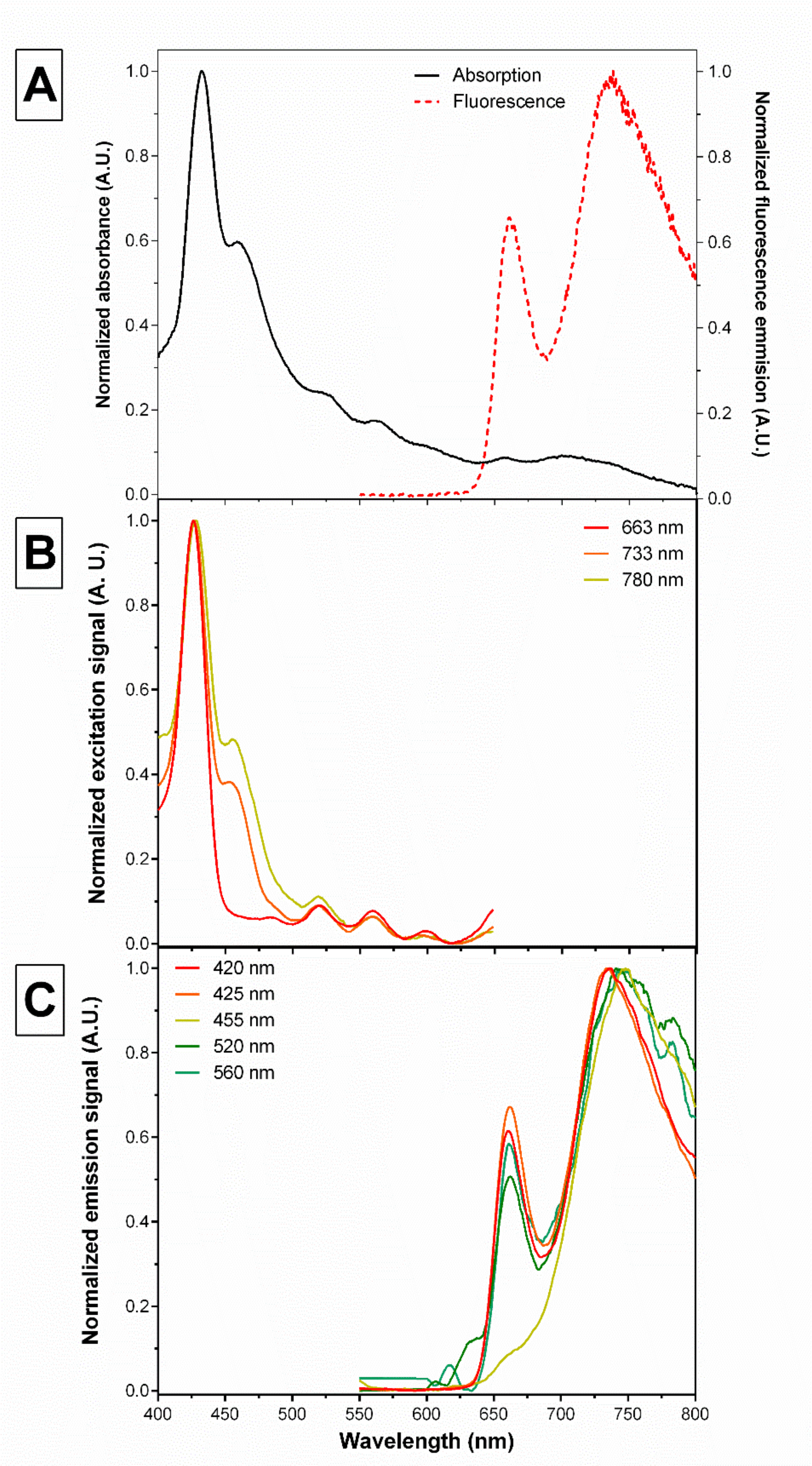

Figure S5. Fluorescent characterization of **THPP@AcLi** 1 μM. (A) UV-vis absorbance (black) and fluorescence emission (red dashed lines) spectra; (B) Excitation spectra at different wavelengths; (C) Emission spectra at different wavelengths. Recorded at PB 0.1 M pH 7, room temperature, λ_{Ex} = 425 nm.

6. Fluorescent quantum yield of THPP@AcLi as a function of pH

Table S6. Φ_F for **THPP@AcLi** 3 μM at different pH, using **TPP** in toluene as a standard ($\Phi_F = 0.11$).

pH	Φ_F
2	0.0016
3	0.0014
4	0.0014
5	0.0014
6	0.0015
7	0.0015
8	0.0014
9	0.0015
10	0.0016

7. PACT effect of @AcLi

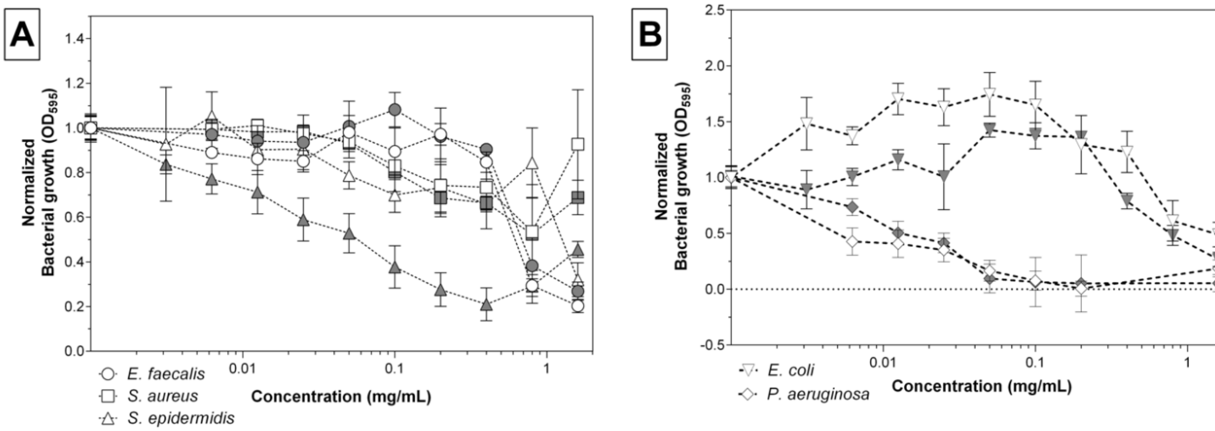

@AcLi nanoparticles were tested against three Gram-positive (*S. aureus, S. epidermidis* and *E. faecalis*) and two Gram-negative bacteria (*E. coli* and *P. aeruginosa*) (Figure S7.1). As the density of nanoparticles was high, results obtained at higher concentrations were difficult to analyze, as nanoparticles, as **@AcLi** also absorb at 600 nm. Nevertheless, the obtained data presents evidence of the lack of photodynamic effect of **@AcLi** against any bacterial strain, as there's not a significant decrease on the bacterial growth when bacteria are exposed to light and **@AcLi** (Table S7.1). However, the presented data seems to indicate that **@AcLi** exerts a non-light-driven bacteriostatic effect, as a decrease on the bacterial growth is observed,in both light and dark conditons. Other researchers have indicated that lignin has an antibacterial effect (Kaur, Uppal and Sharma, 2017; Yang *et al.*, 2018), but no similar evaluations were found in the literature. Interestingly, at some cases (Table S7.1, *S. aureus, E. coli* and *P. aeruginosa)*, the bacteriostatic effect was more evident for the dark controls than for the light irradiated samples. When data was analyzed we found statistical difference between dark and light conditions (P value < 0.05), for *S. aureus* and *E. coli*. Interestingly, in the case of *S. epidermidis* we observed an inversion of the growth tendency at the highest concentrations, while we observe a minor growth in the remaining concentrations (Figure S7.1A). Thus, in general, the presence of lignin into the dark results more toxic than the combination of lignin and light irradiation. As the only difference between the dark and light samples is precisely light irradiation, light seems to be mitigating the bacteriostatic effect of @AcLi. Light irradiation is done at room temperature, with the dark plates covered and away from the light source. As light irradiation is done during one hour, the temperature increases slightly (1 - 2 °C), which may propitiate bacterial growth. Nevertheless, at irradiation time, bacteria are deprived from culture media and thus, bacteria should be able to use lignin as a carbon source in order to continue growing. It has been previously addressed that lignin can be degraded by Proteobacteria, Actinobacteria and Firmicutes, with biodegradation of lignin resulting in a widening research area (Xu *et al.*, 2019). Nevertheless, lignin degrading activity is usually found in lignin-rich environments. Although interesting, the usage of lignin as the sole carbon source is far from the scope of this research but should be noted for future investigations. **Figure S7.1**. Bacteriostatic effect of **@AcLi** under light irradiation (4.16 J/cm^2, white symbols) or dark incubation (dark symbols), against A, three different Gram-positive bacteria and B, two Gram-negative bacteria, *E. coli* and *P. aeruginosa*.

Table S7.1. Comparison of bacterial growth, measured as OD_{600}, after light irradiation (4.16 J/cm^2) or dark incubation, with **@AcLi**. Data was analyzed with a Two-way ANOVA, with a Sidak's multiple comparisons test, statistical differences are considered when the adjusted P value is lower than 0.05.

Strain	Concentration (mg/mL)	Bacterial growth		Adjusted P value
		Light	Dark	
E. faecalis	1.6	0.2027 ± 0.0294	0.2683 ± 0.0405	0.9330
S. aureus	1.6	0.9266 ± 0.2444	0.6883 ± 0.0773	0.0317
S. epidermidis	1.6	0.2850± 0.0356	0.4555 ± 0.0358	0.1911
E. coli	1.6	0.5618 ± 0.0799	0.3182 ± 0.0331	0.0273
P. aeruginosa	1.6	0.1737 ± 0.0886	0.0534 ± 0.0796	0.5298

When bacterial growth results were compared with results from bacterial survival, it was observed that neither a photodynamic or a bactericidal effect was observed when bacteria were exposed to 1.6 mg/mL of **@AcLi**, considering a bactericidal effect when the bacterial survival is lower than 0.1% (Figure S7.2). In conclusion, **@AcLi** may work as a mild bacteriostatic at 1.6 mg/mL, but this effect is not related to the presence of light. There's small evidence that @AcLi could be used as carbon source, but these observations would need further experiments that escape the scope of this study. Is likely that the bacteriostatic effect at 1.6 mg/mL is due to the surfactant properties of lignin. Nevertheless, when loaded with a photosensitizer, as **THPP**, acetylated lignin nanoparticles work as a water-dispersible photosensitizing system, with low chemotoxicity, without any significant contribution of toxicity by @AcLi.

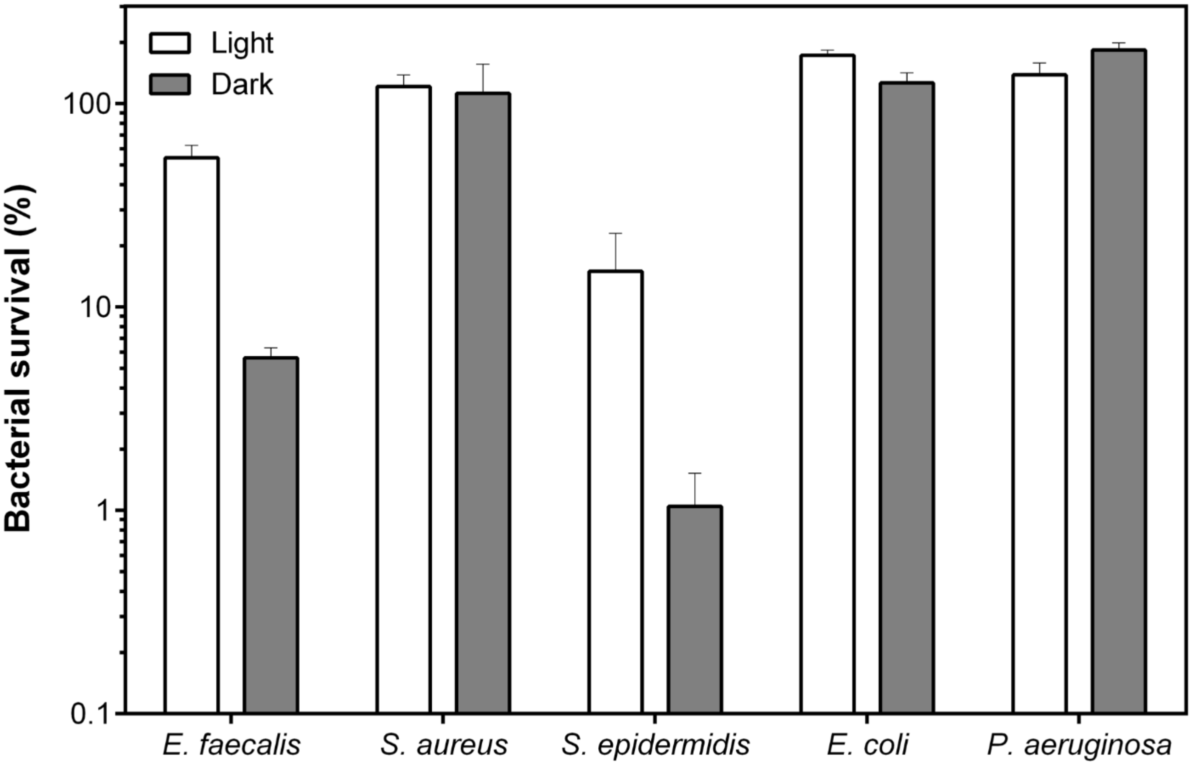

Figure S7.2. Bacterial survival of several bacterial strains with **@AcLi** 1.6 mg/mL treatment, after light irradiation (4.16 J/cm^2) or dark incubation; no differences are found between the light and dark treatment (Two-way ANOVA, Sidak's multiple comparisons test $P > 0.05$).

8. Bacterial growth of five bacterial strains after THPP@AcLi PACT

Table S8. Comparison of bacterial growth, measured as OD_{600}, after light irradiation or dark incubation, with **THPP@AcLi**. Data was analyzed with a Two-way ANOVA, with a Sidak's multiple comparisons test, statistical differences are considered when the adjusted P value is lower than 0.05.

Strain	[**THPP**] in **THPP@AcLi** (μM)	Bacterial growth		Adjusted P value
		Light	Dark	
E. faecalis	0.64	0.0843 ± 0.0094	0.8595 ± 0.0230	< 0.0001
S. aureus	2.56	0.1592 ± 0.0084	0.5874 ± 0.0288	< 0.0001
S. epidermidis	0.078	0.1423 ± 0.0390	0.6274 ± 0.0620	< 0.0001
E. coli	50	1.1981 ± 0.0101	1.2123 ± 0.0111	0.9768
P. aeruginosa	50	0.0247 ± 0.0101	0.0283 ± 0.0122	> 0.9999

9. Bacterial survival of three Gram positive strains after THPP@AcLi PACT

Table S9. Comparison of bacterial survival after light irradiation or dark incubation, with **THPP@AcLi**. Data was analyzed with a Two-way ANOVA, with a Sidak's multiple comparisons test, statistical differences are considered when the adjusted P value is lower than 0.05.

Strain	[**THPP**] in **THPP@AcLi** (µM)	Bacterial survival (%)		Adjusted P value
		Light	Dark	
S. aureus	2.56	0.0739 ± 0.0060	43.0662 ± 3.0837	< 0.0001
S. epidermidis	2.56	0.2874 ± 0.1775	56.65854 ± 4.9189	< 0.0001
E. faecalis	1.28	0.0148 ± 0.0111	39.7717 ± 5.1293	< 0.0001

References

Kaur, R., Uppal, S. K. and Sharma, P. (2017) 'Antioxidant and Antibacterial Activities of Sugarcane Bagasse Lignin and Chemically Modified Lignins', *Sugar Tech*. Springer India, 19(6), pp. 675–680. doi: 10.1007/s12355-017-0513-y.

Marchand, G. *et al.* (2018) 'Acetylated Lignins: A Potential Bio-Sourced Photosensitizer', *ChemistrySelect*, 3(20), pp. 5512–5516. doi: 10.1002/slct.201801039.

Qian, Y. *et al.* (2014) 'Reaction-free lignin whitening via a self-assembly of acetylated lignin', *Industrial and Engineering Chemistry Research*, 53(24), pp. 10024–10028. doi: 10.1021/ie5010338.

Schaberle, F. A. (2018) 'Assessment of the actual light dose in photodynamic therapy', *Photodiagnosis and Photodynamic Therapy*. Elsevier, 23(June), pp. 75–77. doi: 10.1016/j.pdpdt.2018.06.009.

Xu, Z. *et al.* (2019) 'Recent advances in lignin valorization with bacterial cultures: microorganisms, metabolic pathways, and bio-products', *Biotechnology for Biofuels*, 12(1), p. 32. doi: 10.1186/s13068-019-1376-0.

Yang, W. *et al.* (2018) 'Valorization of Acid Isolated High Yield Lignin Nanoparticles as Innovative Antioxidant/Antimicrobial Organic Materials', *ACS Sustainable Chemistry & Engineering*, 6(3), pp. 3502–3514. doi: 10.1021/acssuschemeng.7b03782.
